# Opposing community assembly patterns for dominant and nondominant plant species in herbaceous ecosystems globally

**DOI:** 10.1002/ece3.8266

**Published:** 2021-11-22

**Authors:** Carlos Alberto Arnillas, Elizabeth T. Borer, Eric W. Seabloom, Juan Alberti, Selene Baez, Jonathan D. Bakker, Elizabeth H. Boughton, Yvonne M. Buckley, Miguel Nuno Bugalho, Ian Donohue, John Dwyer, Jennifer Firn, Riley Gridzak, Nicole Hagenah, Yann Hautier, Aveliina Helm, Anke Jentsch, Johannes M. H. Knops, Kimberly J. Komatsu, Lauri Laanisto, Ramesh Laungani, Rebecca McCulley, Joslin L. Moore, John W. Morgan, Pablo Luis Peri, Sally A. Power, Jodi Price, Mahesh Sankaran, Brandon Schamp, Karina Speziale, Rachel Standish, Risto Virtanen, Marc W. Cadotte

**Affiliations:** ^1^ Department of Physical and Environmental Sciences University of Toronto Scarborough Toronto ON Canada; ^2^ University of Minnesota Saint Paul Minnesota USA; ^3^ Instituto de Investigaciones Marinas y Costeras (IIMyC, UNMdP, CONICET) Mar del Plata Argentina; ^4^ Department of Biology Escuela Politécnica Nacional Quito Ecuador; ^5^ School of Environmental and Forest Sciences University of Washington Seattle Washington USA; ^6^ Archbold Biological Station Venus Florida USA; ^7^ School of Natural Sciences, Zoology Trinity College Dublin Dublin Ireland; ^8^ Centre for Applied Ecology Prof. Baeta Neves (CEABN‐InBIO) School of Agriculture University of Lisbon Lisbon Portugal; ^9^ University of Queensland, School of Biological Sciences ST‐Lucia Qld Australia; ^10^ Queensland University of Technology (QUT) Brisbane Qld Australia; ^11^ Queen's University Kingston Ontario Canada; ^12^ Department of Zoology and Entomology Mammal Research Institute University of Pretoria Pretoria South Africa; ^13^ Ecology and Biodiversity Group Department of Biology Utrecht University Utrecht The Netherlands; ^14^ Institute of Ecology and Earth Sciences University of Tartu Tartu Estonia; ^15^ Department of Disturbance Ecology BayCEER University of Bayreuth Bayreuth Germany; ^16^ Department of Health and Environmental Sciences Xi'an Jiaotong Liverpool University Suzhou China; ^17^ School of Biological Sciences University of Nebraska Lincoln Nebraska USA; ^18^ Smithsonian Environmental Research Center Edgewater Maryland USA; ^19^ Department of Agricutural and Environmental Sciences Estonian University of Life Sciences Tartu Estonia; ^20^ Poly Prep Country Day School Brooklyn New York USA; ^21^ Department of Plant and Soil Sciences University of Kentucky Lexington Kentucky USA; ^22^ School of Biological Sciences Monash University Clayton Vic Australia; ^23^ La Trobe University Bundoora Vic Australia; ^24^ INTA‐UNPA‐CONICET Rio Gallegos Santa Cruz Argentina; ^25^ Hawkesbury Institute for the Environment Western Sydney University Penrith Australia; ^26^ Institute for Land, Water and Society Charles Sturt University Albury NSW Australia; ^27^ National Centre for Biological Sciences TIFR Bengaluru India; ^28^ School of Biology University of Leeds Leeds UK; ^29^ Algoma University Sault Ste. Marie ON Canada; ^30^ Grupo de Investigaciones en Biología de la Conservación, Laboratorio Ecotono INIBIOMA (CONICET‐UNCOMA) San Carlos de Bariloche Río Negro Argentina; ^31^ Environmental and Conservation Sciences, College of Science, Health, Engineering and Education Murdoch University Murdoch Western Australia Australia; ^32^ Ecology and Genetics University of Oulu Oulu Finland; ^33^ Department of Biological Sciences University of Toronto Scarborough Toronto ON Canada; ^34^ Department of Ecology and Evolutionary Biology University of Toronto Toronto ON Canada

**Keywords:** biodiversity, community assembly, evolutionary strategies, grasslands, Nutrient Network, phylogenetic relatedness, species dominance, species nondominance

## Abstract

Biotic and abiotic factors interact with dominant plants—the locally most frequent or with the largest coverage—and nondominant plants differently, partially because dominant plants modify the environment where nondominant plants grow. For instance, if dominant plants compete strongly, they will deplete most resources, forcing nondominant plants into a narrower niche space. Conversely, if dominant plants are constrained by the environment, they might not exhaust available resources but instead may ameliorate environmental stressors that usually limit nondominants. Hence, the nature of interactions among nondominant species could be modified by dominant species. Furthermore, these differences could translate into a disparity in the phylogenetic relatedness among dominants compared to the relatedness among nondominants. By estimating phylogenetic dispersion in 78 grasslands across five continents, we found that dominant species were clustered (e.g., co‐dominant grasses), suggesting dominant species are likely organized by environmental filtering, and that nondominant species were either randomly assembled or overdispersed. Traits showed similar trends for those sites (<50%) with sufficient trait data. Furthermore, several lineages scattered in the phylogeny had more nondominant species than expected at random, suggesting that traits common in nondominants are phylogenetically conserved and have evolved multiple times. We also explored environmental drivers of the dominant/nondominant disparity. We found different assembly patterns for dominants and nondominants, consistent with asymmetries in assembly mechanisms. Among the different postulated mechanisms, our results suggest two complementary hypotheses seldom explored: (1) Nondominant species include lineages adapted to thrive in the environment generated by dominant species. (2) Even when dominant species reduce resources to nondominant ones, dominant species could have a stronger positive effect on some nondominants by ameliorating environmental stressors affecting them, than by depleting resources and increasing the environmental stress to those nondominants. These results show that the dominant/nondominant asymmetry has ecological and evolutionary consequences fundamental to understand plant communities.

## Graphical abstract

We found a prevalent disparity between the dominant and nondominant species (measured as the standardized effect size of the mean nearest taxonomic distance), with the former more clustered than the latter, suggesting a disparity in the mechanisms organizing both groups. We also found several clades more likely to have nondominant species than dominant species, measured as the probability of finding a species of a given clade among the third less abundant species in the sites where that clade occurred. Unexpectedly, many nondominant clades have a large number of species, mainly were comprised of nonwoody species, and often appeared in the phylogeny. Together, these findings suggest dominance and nondominance are conserved and that their differences have ecological consequences.

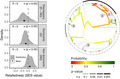

## 1 INTRODUCTION

The relevance of different mechanisms driving species co‐occurrence and co‐existence underlies several of the most important questions in modern ecology (Adler et al., 2007; Chesson, 2000; Lawton, 1999; Palmer, 1994; Vellend, 2010). Resolving these is critical to our understanding of community assembly (Cadotte & Tucker, 2017; Kraft, Adler, et al., 2015) and the impacts of global changes on biodiversity and ecosystem services (Laughlin, 2014; Lavorel & Grigulis, 2012; Seabloom et al., 2013). Co‐existence theories interweave, to some degree, one or more of four mechanisms: restrictions in the movement of individuals or propagules that can arrive in a place; species‐specific responses to environmental conditions; differences among species in the strength of competitive interactions with conspecifics and other species; and stochasticity associated with the previous processes (Chesson, 2000; Hubbell, 2001; Leibold, 1995; Leibold et al., 2004; Vellend, 2010; Weiher et al., 2011; Weiher & Keddy, 1995). Combinations of these mechanisms can explain important ecological patterns, particularly that few species are very abundant in a location (i.e., dominant species), while most species are not (Fisher et al., 1943; McGill et al., 2007). However, the conditions under which a mechanism becomes relatively more or less important than others is still a matter of debate (Cadotte & Tucker, 2017; Jones et al., 2019; Kraft, Adler, et al., 2015; Munoz & Huneman, 2016). Furthermore, it has been seldom explored if species dominance can feed back to affect the relative importance of each of these mechanisms (e.gKhalil et al., 2018; LaPlante & Souza, 2018). Here, we briefly review pertinent theory and evidence supporting an asymmetry in community assembly mechanisms affecting dominant and nondominant species and the mechanisms that drive trait and phylogenetic dispersion patterns then propose a way to test the existence of such asymmetries. Finally, we test that conceptual framework using a global grassland dataset (Borer et al., 2014).

### 1.1 Evidence for different mechanisms driving dominants and nondominant species

Classical community assembly research typically assumes that all species in a given community are subject to similar assembly processes that are revealed by the analysis of community‐wide patterns (Gilbert et al., 2009; e.g., Weiher et al., 2011; Weiher & Keddy, 1995). For instance, communities are often described as being determined mostly by environmental filtering or limiting similarity (for alternative approaches see Chalmandrier et al., 2013; Lortie et al., 2004). However, several conceptual frameworks (e.g., core‐satellite: Hanski, 1982; dominant‐subordinate‐transient: Grime, 1998; facilitation: Brooker et al., 2008; Mariotte, 2014; foundation species: Ellison, 2019; and references therein) and empirical evidence (Lennon et al., 2011; Magurran & Henderson, 2003; Maire et al., 2012; Schöb et al., 2012) suggest fundamental differences in the relative importance of ecological processes between dominant and nondominant plants. Following Magurran (2013), we refer to “abundance” as different ways to measure dominance.

Using a dominant removal experiment in a temperate meadow, Arnillas and Cadotte (2019) tested the prevalence of stochastic vs. deterministic assembly rules in plant communities. In that experiment, as in this study, dominant species (sensu Rabinowitz, 1981) were defined as those that can capture most of the resources in a homogeneous area in which dispersal limitation can be assumed as negligible. Hence, at the beginning of the experiment in five experimental sites, Arnillas and Cadotte (2019) identified the most dominant species and the nondominant species based on cover (per plot), height (per plot), and frequency (among plots). At the end of the experiment, in the plots where dominant species were removed, they identified the nondominant species that became the new dominants (based on cover and height, frequency was not included at the end as it would bias the results in the context of that experiment). By comparing the compositional changes in multiple plots where the dominant species were systematically removed against control plots, they found that the new dominant species behaved more deterministically (decreasing their among‐plot dissimilarity) than the nondominant species that stay as nondominants. That trend indicates that deterministic mechanisms became more important for the originally nondominant species that became dominant, but not for the other nondominant species. Therefore, Arnillas and Cadotte’s (2019) results suggest that determinism increased with dominance, which in turn indicate that the ecological differences between dominant and nondominants have implications at the community level. In this study, we aim to test the generality of this finding across grasslands around the world.

### 1.2 Trait and phylogenetic dispersion patterns: inferring assembly mechanisms

Deterministic (i.e., nonrandom) community processes can generate over‐ or under‐dispersion (clustering) according the community assembly theory (Weiher & Keddy, 1995). Assuming that all species respond to similar rules, community assembly theory conceptualizes the mechanisms that determine the species that co‐exist as successive filters, which determine which species from the meta‐community will be found in a local community (Leibold & Chase, 2017). The first filter, *dispersal limitation*, constrains the species able to reach the community from the species pool in the meta‐community, and it is negatively related to the migration of propagules to the local community. Then, *habitat filtering* restricts resident species to those possessing traits that confer positive fitness within local environmental conditions and that allow these species to outperform species possessing suboptimal traits (e.g., frost tolerance or not in an alpine environment). Finally, *limiting similarity* refers to negative interspecific interactions (e.g., soil nutrient competition) that select for species possessing complementary resource acquisition traits and niche differences allowing them to coexist indefinitely. Deterministic selection acts through habitat filtering and limiting similarity, and both are affected by species traits (Vellend, 2010). Stochasticity, or drift, is often attributed to unexplained variation in species abundances (Vellend et al., 2014). Among other options, stochastic patterns can arise because of stochastic outputs of individual interactions or by deterministic interactions between individuals if functional trait values of each individual are randomly assigned and not associated with species identity. Despite the oft‐articulated logic that these filters occur in sequential order, in reality they are not discrete but rather occur simultaneously and interact (Cadotte & Tucker, 2017).

The outcomes of community assembly processes might be detectable in traits: Strong habitat filtering should imply traits more similar than expected by random (clustering or underdispersion), while limiting similarity should generate the opposite pattern (overdispersion; Weiher et al., 2011). Phylogenetic patterns may contain the imprint of this process, if the species traits governing community assembly are shared by closely related species, providing additional insights into coexistence, as suggested by Webb (2000) (see also Ackerly, 2003; Cavender‐Bares et al., 2004). Although the similarity–relatedness relationship is not always valid (Cadotte et al., 2017; Cavender‐Bares et al., 2009; Gerhold et al., 2015; Münkemüller et al., 2020), it often provides a good first approximation of ecological differences and similarities among species and algorithms that predict trait values will often rely on phylogenies (Schrodt et al., 2015; Swenson, 2020). For example, when habitat filtering is stronger than limiting similarity, co‐existing species will be more closely related than expected by chance and will appear as clustered when analyzed (Gerhold et al., 2015; Webb, 2000). Conversely, if limiting similarity is stronger than habitat filtering, the surviving species will be more distantly related than expected at random (i.e., species will be overdispersed). These predictions are especially true if multiple, independently evolved traits influence these ecological processes (Cadotte et al., 2017; Tucker et al., 2018), and less likely to be true if relatively few traits, especially those that converged evolutionarily, drive ecological processes (all else being equal). Even when trait and phylogenetic community patterns are not completely congruent, phylogeny offers insights into assembly mechanisms that are not captured by measured traits (Bässler et al., 2014; Cadotte et al., 2013, 2019). Given such considerations, clustered and overdispersed phylogenetic patterns in plant communities are evident when (1) species functional similarity is correlated with phylogenetic relatedness, (2) either habitat filtering or limiting similarity is stronger than the other, and (3) dispersal limitation and stochastic processes do not bias or obscure these patterns (Chalmandrier et al., 2013; Gerhold et al., 2015) (Table A1 presents a detailed list of assumptions of this approach).

### 1.3 Signs of dominance asymmetry in phylogenetic and trait dispersal patterns

We hypothesized that dominant and nondominant plant species assemble differently at the local level (i.e., at a scale that includes direct individual interactions and population level dynamics, but without dispersal limitation) because they are likely to interact with the environment in distinct ways. Dominant plant species often capture more sunlight and other resources, outcompeting nondominant species. Local nondominant species could use marginal habitats or conditions, rely on spatial or temporal niche partitioning (e.g., early or late season plants) or perhaps have evolved to utilize or rely on the environmental conditions created by dominant plants, and would thus likely appear to be facilitated by dominant species, especially in harsh, unproductive, or heavily grazed environments (Bertness & Callaway, 1994; Lortie & Callaway, 2006). Further, dominant species could create small spatially heterogeneous patches, increasing the number of niches available for nondominant species (Aarssen et al., 2006). Together, these mechanisms suggest that dominant species are more likely than nondominant species to be influenced by the environment and also more likely to shape surrounding conditions than nondominants, a prediction consistent with the mass‐ratio hypothesis (Grime, 1998). In spite of these clear predictions, these theoretical expectations has not been tested across many site conditions.

Further, we hypothesize that the asymmetry between dominant and nondominant species in their interactions with the environment could generate a disparity in the average phylogenetic relatedness (or distances) among dominant species compared to the phylogenetic distances among nondominant species. For brevity, we refer hereafter to these differing expectations of phylogenetic relatedness for dominant and nondominant species as “relatedness disparity.” Although we interpret patterns based on the assumptions of Webb (2000), alternative interpretations under different assumptions—including the role of facilitation—are presented in Table A1, showing that a relatedness disparity would indicate that either a different mechanism exists for dominant and nondominant species, or that some kind of intrinsic difference among the dominant and nondominant species exists. We refer to the combination of assumptions and observed relatedness disparity as alternative scenarios.

We postulate three possible scenarios based on Webb (2000) assumptions and show their expected relatedness disparity: First, if dominant species strongly compete and deplete most of the resources, limiting similarity should cause the dominant species to be over‐dispersed. The reduction in resources available for nondominants should then act as an additional habitat filter, reducing the phylogenetical dispersion of nondominants to those few groups that can take advantage of the remaining resources. In this scenario, dominant plants should be less phylogenetically related than nondominants (hereafter, positive relatedness disparity). Second, if habitat filtering in any given site constrains the dominant species to those possessing the key traits or ecological strategies optimal for those conditions, and these dominant species not only reduce (but not deplete) local resources but also moderate environmental conditions (especially reducing extreme environmental fluctuations) for nondominant species, we expect dominant species to be more clustered (underdispersed) than nondominant species (hereafter, negative relatedness disparity). Negative relatedness disparity also could occur if the dominant species generate multiple small niches where small nondominant species can thrive (Aarssen et al., 2006). Third, no disparity is expected if stochastic mechanisms prevail at the species level (i.e., neutral model; sensu Hubbell, 2001) or if community assembly processes act similarly on dominant and nondominant species (e.g., if water availability limits dominant species and light limits nondominant species in a dry area both groups will appear as phylogenetically clustered). Similarly, some phylogenetic tree topologies could bias the disparity (e.g., long terminal tips in a balanced tree). Analogous scenarios can be interpreted in terms of trait dispersion, but the trends could be hindered if the available trait information does not adequately represent dominant and nondominant species. In their experimental study, Arnillas and Cadotte (2019) found a pattern consistent with the second scenario: The similarity among plots where dominant species were removed increased when they focused their analysis on the new dominant species, while they found signs of randomness or overdispersion driving other nondominant species.

### 1.4 Exploring sign and drivers of *relatedness disparity*

In this study, we quantified the relatedness disparity associated with dominance in herbaceous ecosystems using a large database describing a large number of grasslands around the world (where each site is represented by at least 30 plots of 1m^2^), and explored potential drivers of that disparity (Borer et al., 2014). Because dominance in each grassland is locally determined, the magnitude and sign of the relatedness disparity can change from one site to another, even if the same species occupy both sites. Hence, we treat each site as an independent observation. Further, herbaceous ecosystems such as grasslands typically possess at least one dominant graminoid species (often either a grass or a sedge), and strong limiting similarity among dominants should decrease the probability of dominance by more than one graminoid, increasing the phylogenetic dispersion of dominant species in each site. Conversely, strong habitat filtering of dominants should increase the odds of other dominant graminoid species being present (like asymmetric competition in Mayfield & Levine, 2010), reducing the phylogenetic dispersion of the dominant species. In other words, we aim to explore relatedness patterns of species *sharing* dominance and of those *sharing* nondominance.

First, to test if any of the three scenarios previously described (positive, negative, or null relatedness disparity) was more likely to occur in grasslands, we determined if dominant and nondominant species were similarly assembled by measuring their relatedness disparity in different sites. We also explored if the strength of relatedness disparity was driven primarily by phylogenetic relatedness among dominant or nondominant species and compared these trends with the trends observed in traits for which there were sufficient samples. Second, since disparity is locally defined, we looked for plant lineages—branches in the phylogeny—consistently categorized as either dominant or nondominant across different sites. Assuming a neutral pattern as a null hypothesis, we expected a similar number of species from of each lineage, including graminoids, in each dominance category. Finally, we tested some potential drivers that could affect relatedness disparity for dominant and nondominant species. Specifically, we tested whether disparity trends among sites were related to tree topology, aboveground biomass (or drivers of productivity such as climate, human management, legume biomass as a proxy of nitrogen fixation), or—as the study focused on grasslands—differences in graminoid biomass. We also considered our results in the context of seven key assumptions underlying the phylogenetic approach (Gerhold et al., 2015; Table A1).

## 2 METHODS

### 2.1 Data sources

#### 2.1.1 Site‐level data

We analyzed data collected as part of the Nutrient Network, a distributed, collaborative project in the world's grasslands (hereafter NutNet, http://www.nutnet.org; Borer et al., 2014). For this study, we quantified cover and biomass in at least 30 unmanipulated plots per site during the peak growing season (each site may have been sampled in a different year, database accessed on 2018‐12‐20). Each plot was 5 × 5 m and was divided into 4 2.5 × 2.5 m subplots. In one subplot, we measured the cover of each species in a 1 × 1 m quadrat. Cover can sum to more than 100% because of multi‐layer canopies. We also cut aboveground biomass from two 0.1 × 1 m strips adjacent to the cover quadrat, sorted the live biomass (current year's growth) to functional group (e.g., graminoids, forbs, legumes, mosses), dried it to a constant mass, and weighed it to the nearest 0.01 g. For most sites, the plots were in homogeneous areas where the distance between contiguous plots was <5 m.

We calculated vascular species dominance for each site as the mean species total percent cover across all plots (*cover*, including zero values). We performed similar analyses using the proportion of plots where the species was present (*frequency*) and the mean species cover in these plots (*cover presence*‐*only* or *cover PO*). The three variables are related, as *cover* = *frequency* × *cover PO*, and might capture different ways in which a species can dominate an area (e.g., dispersal limited competitive dominants would have low‐frequency and high‐cover PO).

We included categorical site management descriptors (site restored or anthropogenically created, site under active managed burning regime, site regularly grazed by herbivores) and biomass‐derived measurements to identify variables that could explain global differences in relatedness disparity (Table A2 in Appendix 1). We calculated the proportion of living biomass of graminoid species to estimate graminoid prevalence. We summed plot level functional group biomass, and we used legume aboveground biomass as an indirect estimate of potential nitrogen fixation. We used only data from sites in which functional group biomass was measured in the same year as cover data. Climatic information was obtained from WorldClim 2 (Fick & Hijmans, 2017).

Our final dataset included 78 sites—each site with at least 30 plots with species cover data. A subset of 63 of these sites had complete site descriptors and biomass information by functional group (Figure A1 in Appendix 1). All sites had 13 species or more (75% had at least 21 species), and three or more graminoids (75% had at least seven graminoids).

All analyses were done in R version 3.4.2 (R Core Team, 2017).

#### 2.1.2 Phylogenetic information

We adapted the Qian and Jin (2016) phylogeny and methodology to create a phylogenetic tree with every vascular plant species present in the NutNet dataset (Borer et al., 2014) by adding species absent from Qian and Jin’s (2016) tree to a congeneric species present in the tree (46% of the observed species). Where no congeneric species was available, we used the family node (4.3% of the observed species, details in Appendix 2; see also Li et al., 2019). The impact of missing data was likely minimal, however. In particular, in 7.3% of the 2437 genus‐site combinations was a species absent in the phylogeny that had one or more congeneric species in the same site. We adapted the phylogeny using the packages APE (Paradis et al., 2017) and apTreeshape (Bortolussi et al., 2012).

To assess the role of different phylogenetic topologies in the observed relatedness patterns, we pruned the tree to the species present in each site and estimated the number of species, Faith's phylogenetic diversity (hereafter PD) as a measure of phylogenetic history (Faith, 1992), and three tree topology indices (Table A2).

### 2.2 Are dominant and nondominant species similarly assembled?

To assess whether dominant species were more closely related to one another than nondominant species are to one another (i.e., dominance disparity in relatedness or simply dominance disparity), we split the species found at each site into three equally sized groups or partitions (i.e., dominant, intermediate, and nondominant) according to the species rank cover values. For this analysis, we used the dominant and nondominant partitions as they represent the extremes of the dominance spectrum. Even though forcing a symmetric partition might not be ecologically meaningful, we used this partition as it requires fewer assumptions and previous work has shown that it provides similar results to other ways of partitioning communities (Umaña et al., 2017). We compared different partition approaches and show that they all correlate (Supplementary information SI1).

For each site, we calculated the mean nearest taxonomic distance (MNTD, the average phylogenetic relatedness between a species and its closest relative in a site) for dominants (D_MNTD_) and nondominants (ND_MNTD_) to test for the asymmetries in phylogenetic dispersal between dominants and nondominants. By comparing phylogenetic dispersal patterns among partitions of the same site, we can be certain that dispersal limitation (i.e., species capability to arrive to each site) will not affect the observed patterns. We built random expectations by randomly swapping the tree tips 999 times without weights, which is equivalent to randomly assigning each species to each dominance partition and measuring the MNTD of the random sample. We did not use weights during the sampling because coverage was already used to divide the community. This algorithm assesses if the species in a dominance partition are more (or less) closely related among them than expected from a random draw of the species occupying the site. We estimated the dominant and nondominant relatedness as the standardized effect size (SES) of their respective MNTD (i.e., dominant partition: D_SES.MNTD_ = (D_MNTD_ – MNTD_MEAN_)/MNTD_SD_, and nondominant partition: ND_SES_._MNTD_ = (ND_MPD_ – MNTD_MEAN_)/MNTD_SD_), where MNTD_MEAN_ and MNTD_SD_ are the mean and the standard deviation of the observed and randomly generated MNTD values together, respectively, for both the dominant and the nondominant partition). Because of this normalization, the expected variance of each SES is 1. For each site, our SES estimates approach zero when the species in a partition are random relative to the species phylogeny, negative if these species are clustered (more closely related than expected), and positive if they are overdispersed (more distantly related than expected). We measured the relatedness disparity (Δ_SES.MNTD_) at each site as the difference between the relatedness of the dominant partition (D_SES.MNTD_) and the nondominant partition (ND_SES.MNTD_). A positive relatedness disparity (Δ_SES.MNTD_ = D_SES.MNTD_ ‐ ND_SES.MNTD_ >0) indicates that dominant species are more distantly related than nondominants, while a negative relatedness disparity indicates the opposite trend.

Following our three scenarios presented previously, our main target was to explore if relatedness disparity values (Δ_SES.MNTD_) were different from zero locally and globally. We also tested dominant relatedness (D_SES.MNTD_) and nondominant relatedness (ND_SES.MNTD_) to explore which of these components determines the disparity patterns. Locally, relatedness values more extreme than ±1.96 *s* would indicate enough evidence that the site phylogenetic dispersion is not random, where *s* is the expected standard deviation (1 for D_SES.MNTD_ and ND_SES.MNTD_, and $\sqrt{2}$ for Δ_SES.MNTD_). For the global tests, we took each site as an independent observation representing D_SES.MNTD_, ND_SES.MNTD_, and Δ_SES.MNTD_ values of grasslands around the world, and used either Kolmogorov‐Smirnoff goodness‐of‐fit test or Wilcoxon signed rank test (both assuming µ = 0) depending if the relatedness values were normally distributed or not (details in Appendix 2).

We ran similar analyses to calculate the dominance disparity using the species ranking determined by the other two dominance metrics (frequency and cover presence‐only, alternative ways to define dominant species are presented in Supplementary Information SI1). Also, we repeated these analyses using mean phylogenetic distance (MPD) and obtained Δ_SES.MPD_, D_SES.MPD_ and ND_SES.MPD_. MPD is the average phylogenetic relatedness of all pairs of species. Because MPD includes all species pairs, MPD is more sensitive to the basal structure of the tree, while MNTD is more sensitive to the branching of the tips of the phylogeny (Cadotte & Davies, 2016).

Finally, we compiled trait data for our species in the Flora of North America, TRY, BIEN and Pladias databases (Gleason & Cronquist, 1991; Kattge et al., 2020; Maitner et al., 2018; Pladias; https://pladias.cz, respectively). We ran analyses of trait dispersion analogous to the ones done for phylogenetic dispersion for the four traits with sufficient information for dominant and nondominant species: leaf dry mass per leaf fresh mass (615 species, 34%), leaf nitrogen content per leaf dry mass (676, 37%), seed mass (1039, 57%), and whole plant height (906, 50%). Gaps in the trait information (Table SI3) precluded extensive trait analyses. Details are included in Supplementary Information SI2.

### 2.3 Are certain lineages more likely to be either dominant or nondominant?

Relatedness disparity is a site‐specific measure, and given that species pools change from site to site, we were interested in assessing if similar species were consistently present among partitions across sites. Because of the low probability of finding the same species in sites around the world, we used phylogenetic beta‐diversity metrics that compare the proportion of phylogenetic branches shared between sites (Baselga et al., 2017; Leprieur et al., 2012). Therefore, our test assessed the consistency of *lineages* among partitions across sites. A lineage is any monophyletic group of phylogenetic branches originating from a single ancestral node (also referred to as a clade), regardless of the taxonomic designation (e.g., genus, family). For each partition, we estimated the Sørensen‐derived phylogenetic multisite and pairwise‐dissimilarity indices and tested if the beta‐diversity of each partition was similar to a random expectation (random model explained below). We also obtained the nestedness‐ and turnover‐fractions of both indices, to assess whether dissimilarity of each partition increased by loss of certain branches of the phylogeny (nestedness) or by their replacement (turnover; Baselga, 2010). For these analyses, we built 499 random datasets by shuffling the species among the three partitions in each site (dominant, intermediate and nondominant), with all species having an equal probability of being in any partition, and estimated the beta‐diversity indices among sites for each randomly generated dataset. As the random distribution of the three partitions for each index was very similar, we combined them and compared each observed phylogenetic dissimilarity value against the combined 1497 randomly generated observations. The null hypothesis was that all species and lineages are equally likely to be in any dominance partition at a global scale. To control for potential biogeographic bias, we repeated the analysis removing data from Australia, which tends to be unique in several biogeographic aspects, and North America, where most of the sites were located.

To identify which lineages were more likely to be dominants, while controlling for global lineage occurrence differences, we counted the number of sites in which any species of that lineage was dominant and compared that value with the total number of species of that lineage in any site. We assumed that the probability that any taxon in any site being in each dominance partition was identical (1/3) and ran a binomial test in each branch with 10 or more counts in that lineage. We repeated the analysis with the intermediate and nondominant partitions. We also did a similar analysis at the genus level, including all genera regardless of the total number of counts.

### 2.4 Are there environmental conditions, topological characteristics of the phylogeny, or biogeographic aspects that drive relatedness disparity?

We explored potential drivers of relatedness disparity differences among sites. Particularly, we focused on graminoid prevalence (i.e., the proportion of site biomass composed by graminoids), tree topology, site aboveground biomass, climatic conditions, geographic location, and site management. Given that most sites have at least one dominant graminoid, we started the analysis assuming that graminoid prevalence mediates any differences in relatedness disparity (Δ_SES.MNTD_), dominant relatedness (D_SES.MNTD_), and nondominant relatedness (ND_SES.MNTD_). Therefore, any effect of another independent variable on any of these three relatedness metrics will be detected as additional to the chances caused by graminoid prevalence.

We ran preliminary backwards‐stepwise regressions on linear models (using AIC as the model selection criterion) to identify the subset of variables (see Table A2) that were more likely to be important in explaining Δ_SES.MNTD_, D_SES.MNTD_, and ND_SES.MNTD_. For each of these three variables independently, we assumed that (i) the variables identified with the previous step were predictors of graminoid prevalence and (ii) graminoid prevalence was the only (linear) predictor of each of the three variables. We combined these two assumptions and built three models in which we tested whether Δ_SES.MNTD_, D_SES.MNTD_ and ND_SES.MNTD_, independently, are d‐separated (controlled by graminoid prevalence) from their respective predictors previously identified using the backwards‐stepwise selection approach. We used the piecewiseSEM package (Lefcheck, 2016) to perform the d‐separation tests. Similar analyses were done with Δ_SES.MPD_, D_SES.MPD,_ and ND_SES.MPD_. We assessed the amount of information provided by each group of variables as the difference between the final model *R* ^2^ and the same model without the variables in that model that corresponded to the different categories described in Table A2.

## 3 RESULTS

### 3.1 Are dominant and nondominant species similarly assembled?

**FIGURE 1:**
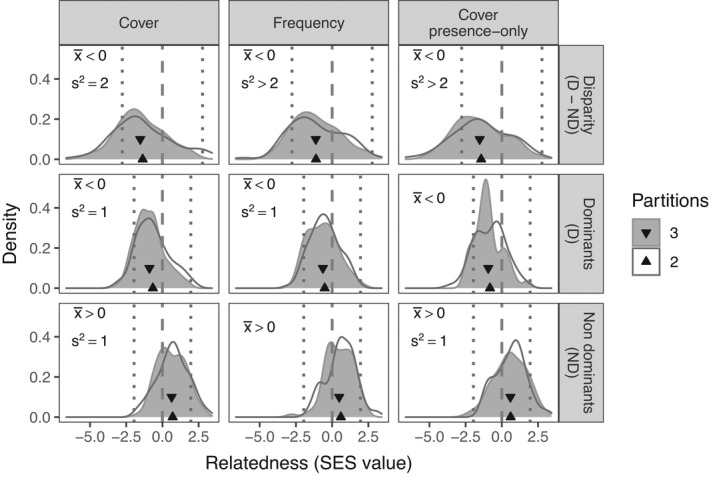
Global and local tests of relatedness disparity between dominants and nondominant plants, and the relatedness of these partitions. Each row represents a relatedness value and the columns represent different ways to measure the dominance of the species. We partitioned the community into two (clear) and three (gray) partitions (each partition with a similar number of species) and plotted the density of sites with the respective relatedness value. The relatedness in each site and partition is the standardized effect size of the mean nearest taxonomic distance (MNTD). For the local tests, vertical dotted lines represent the limit for an independent site to be considered equal to zero. Therefore, the areas beyond the dotted lines indicate the proportion of sites with enough evidence by themselves of a nonrandom assortment. For the global test, triangles represent the mean value for each partition, vertical dashed lines represent zero (which indicates random assortment), and the letters in the top‐left corner indicate if the global phylogenetic dispersion was different from zero or not. For that test, the distribution for the three partitions was tested for normality first. When non‐normal, we tested whether the mean ($\bar{x}$) was lower or higher than 0. If normal, we also tested if the variance (s^2^) was lower or higher than the expected variance (2 for disparity, 1 for relatedness). All tests were done at *p* < .05

By measuring relatedness disparity (Δ) as the difference between the standardized effect size (SES) of the mean nearest taxonomic distance (MNTD) of the top third most dominant species (D) and the bottom third least dominant species at each site (ND), we found negative relatedness disparity globally (Δ_SES.MNTD_ = −1.53 ± 1.62 [mean ± *SD*], 78 sites, Kolmogorov–Smirnov two‐sided test's *p*‐value < .001, gray area shows density distribution and triangles pointing down show the means in Figure 1). In this case, dominant species were more closely related than expected by chance (D_SES.MNTD_ = −0.90 ± 1.01, *p *< .001). Conversely, nondominant species were overdispersed (ND_SES.MNTD_ = 0.64 ± 0.98, *p *< .001). Besides these global trends, local disparity trends were strong enough in some sites that if we were to run the analysis in individual sites, the SES values will indicate a disparity exist (areas beyond the dotted lines in Figure 1). These results were consistent for frequency, mean cover of the plots where species were present only, and overall mean cover, the latter including the effect of the first two (Figure 1, in all cases, *p* < .05). The results were also robust to the use of two instead of three partitions (Figure 1) and to other partition approaches (Supplementary Information SI1). Further, we found similar results using mean phylogenetic distance (MPD) instead of MNTD: Δ_SES.MPD_ and D_SES.MPD_ were both negative, but ND_SES.MPD_ was indistinguishable from zero (see Figure A2).

Traits provided a similar picture to the phylogenetic analyses: For each of the four focal traits, dominant plants tended to be more similar to one another than nondominants using at least one dominance metric (Figure SI3). Furthermore, no combination of trait and dominance metric showed evidence of dominant species more dissimilar than nondominants. Using cover, an average of only 37 sites (47% of total, range between 19% and 57%) had enough information to do an adequate analysis (Table SI4), and the nondominant species were less thoroughly sampled than the dominant ones (Table SI3).

### 3.2 Are certain lineages more likely to be either dominant or nondominant?

**FIGURE 2:**
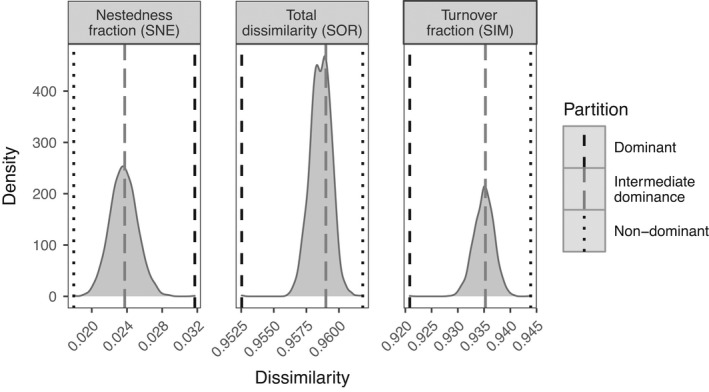
Phylogenetic dissimilarities among sites when each site is partitioned into dominant, intermediate dominance and nondominant species, each partition with a third of the species. The total phylogenetic dissimilarity is measured as the multisite Sørensen (SOR), and decomposed in turnover (SIM) and nestedness (SNE) fractions. Dashed lines represent the observed values when species dominance is assigned based on mean cover per plot, while the density curves represent the probability of a given dissimilarity value if the species were randomly distributed in the three partitions

We measured the spatial phylogenetic dissimilarity patterns and estimated its components—turnover and nestedness fractions—for each partition. Phylogenies between sites were consistently dissimilar (Figure 2), mostly because of large species spatial turnover, as expected because of the global scope of the study. Despite the large species turnover, sites shared lineages of dominant species more often than expected by chance (*p* < .001, Figure 2a), mostly because some lineages were present more commonly than expected by random assembly (i.e., turnover fraction of the total beta diversity smaller than chance). Conversely, the nestedness fraction of dissimilarity of dominants was larger than expected by chance (*p* < .001, Figure 2b). When the nestedness fraction is measured using species, a large value indicates a strong reduction in the number of species. By extension, a large nestedness fraction in this phylogenetic beta‐diversity index indicates that, at sites with fewer species, the species present belonged to fewer lineages than expected by random assembly. The pattern was reversed for nondominant plants (i.e., compared to a random distribution, the observed values indicate more dissimilar lineages around the world, several lineages appearing in different sites with the lineages that become absent more scattered across the phylogeny than expected by random chance). The intermediate partition was indistinguishable from random assembly. These patterns were robust to the exclusion of Australian or North American sites, and to the use of multisite and mean pairwise indices (Figure A3).

**FIGURE 3:**
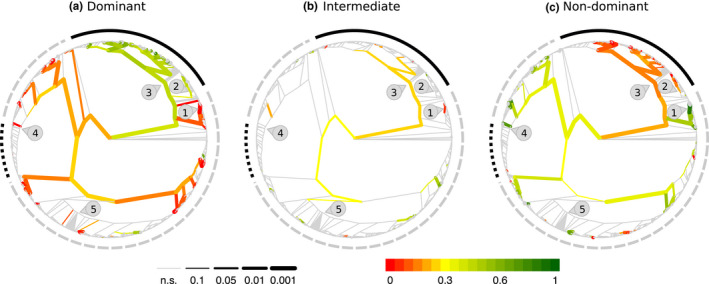
Phylogenetic tree of species observed in the experiment showing the probability of a lineage to be dominant, intermediate or nondominant. The dominance partitions were determined at each site independently, with a third of species in each site in each of the partitions. A gray edge indicates that the lineage was present in less than 10 sites (not enough cases to take a decision) or that the proportion is not different than 1/3 (*p* > .1). Red colors indicate proportion lower than expected, and green colors proportion higher than expected. Edge width indicates if the proportion is significantly different from 1/3. Groups symmetrically distributed in the three dominance categories have gray edges in the three trees. Outside arcs indicate functional groups: graminoids (black solid), legumes (black dotted), any other functional group, mainly forbs (gray dashed). Numbers indicate some families: 1. Orchidaceae, 2. Cyperaceae, 3. Poaceae, 4. Fabaceae, 5. Asteracea

In contrast to the hypothesis that grasses would be equally present in all the dominance partitions within each site, grasses and sedges were more likely to occur in the dominant partition and less likely to occur in the intermediate or nondominant partitions (Figure 3a, details in Figure A4). Among the 113 genera of grasses, 9 were more frequently associated with dominant species (e.g., *Bromus*, *Elymus*, *Poa*, *Panicum*, *Sporobolus*), and of the 15 genera of sedges, only *Carex* (family Cyperaceae) also were frequently dominant (Table A3a). Among nongraminoids, *Solidago* and *Hypochaeris* (fam. Asteraceae) were likely to contain dominant species (*p* < .05). *Lespedeza* (fam. Fabaceae), *Phlox* (fam. Polemoniaceae), and *Baccharis* (fam. Asteraceae) were more associated with dominant species (*p* < .05) but were present in very few sites (<10). We found a similar trend in the lineage of the family Acanthaceae but the low number of sites deterred the identification of the genera.

More than a dozen different lineages were associated with nondominant species more often than expected by chance (*p* < .05, Figure 3c). In contrast to the strong dominance of the graminoid lineage, nondominant lineages covered a larger portion of the phylogenetic tree. Among the monocots, several lineages in the orders Liliales and Asparagales (e.g., orchids) were more often nondominants, although the small number of species sampled from each genus made trends at the genus level unclear (Tables A3c and A4). Dicot lineages more likely to be identified as nondominants included the genera *Brassica* (Brassicaceae) and *Geranium* (Geraniaceae). As with the orchids, the list of genera did not always represent the lineages that were identified as more likely to be nondominants (Figure A5, Table A3c).

### 3.3 Are there environmental conditions, topological characteristics of the phylogeny, or biogeographic aspects that drive the relatedness disparity?

We explored potential drivers (Table A2) of the differences in the relatedness disparity (Δ_SES.MNTD_, Δ_SES.MPD_), dominant relatedness (D_SES.MNTD_, D_SES.MPD_), and nondominant relatedness (ND_SES.MNTD_, ND_SES.MPD_) observed at the global scale using stepwise regression and found that graminoid prevalence (observed range: 0.14–1) by itself consistently decreased disparity and dominant relatedness using MNTD and MPD (*p* < .05), indicating that two or more species of the same lineages shared dominance in sites with more graminoid biomass. However, its effect on nondominant relatedness was marginal with MNTD (*p* < .1) or negligible with MPD (Table A5). Besides graminoid prevalence, sites varied widely in climate (e.g., average annual temperature: −8–27°C, annual precipitation: 216–2224 mm) and community parameters (e.g., species richness: 13–94, see also Table A2). Temporal distribution of nodes in the phylogeny—represented by the Gamma index—was important for MPD metrics, probably driven by the fact that graminoids tended to be dominant despite they diversified more recently than other lineages. Richness intensified dominant clustering but had no effect on nondominants. Together, these predictors explained between 22 and 29% of the relatedness disparity, dominance relatedness, and nondominant relatedness measured with MNTD related metrics, and between 30 and 44% of the MPD ones (Table 1).

**TABLE 1:** Best models describing the slopes between relatedness disparity (Δ_SES.MNTD_, Δ_SES.MPD_), dominance relatedness (D_SES.MNTD_, D_SES.MPD_), and nondominant relatedness (ND_SES.MNTD_, ND_SES.MPD_) with site level descriptors. Relatedness measured using mean nearest taxonomic distance (MNTD) and mean phylogenetic distance (MPD). Site level descriptors include location, climate, management, tree topology, and aboveground biomass. Last two rows indicate the coefficient of determination (*R* ^2^) and the *p*‐value of the residual normality test done using the Shapiro–Wilk test

Predictor	Δ_SES.MNTD_	D_SES.MNTD_	ND_SES.MNTD_	Δ_SES.MPD_	D_SES.MPD_	ND_SES.MPD_
(Intercept)	1.458	2.317*	0.653	3.931^†^	3.110^†^	1.463
Elevation		0.0005*	0.0002		0.0003^†^	0.0003^†^
Annual precipitation^a^						0.401
Daily temperature range	−0.116^†^	−0.121*				
Mean annual temperature		0.041	0.039^†^			0.045^†^
Mean diurnal temperature range				−0.165*	−0.091	
Temperature annual range				0.081**	0.029	−0.026
Anthropogenic origin			0.585*	−1.023*		0.598^†^
Grazed	0.687		−0.631^†^	0.983^†^	0.726*	
Burned					−0.633	
Recent				−5.146^†^	−3.17	
Gamma statistic^b^				−0.246*		0.237*
Richness		−0.024**			−0.017*	
MPD						−0.015^†^
MNTD		−0.011^†^				0.019*
Graminoid prevalence	−2.504***	−1.355*	1.207^†^	−2.043*	−1.987**	
Biomass^a^			−0.276			−0.395^†^
*R* ^2^	.221	.275	.287	.376	.439	.300
Normality of the residuals (*p* value)	.198	.553	.102	.727	.066	.050

Notes

Final model include only variables kept after the AIC backwards‐step variable selection process. List of variables can be found in Table A2.

^a^ Log‐transformed.

^b^ Gamma statistic represents the temporal distribution of nodes in the phylogeny (negative values: deeper nodes; positive values: shallower nodes). All regressions were done with 62 observations. Residual normally was assessed using Shapiro–Wilk test. Significance: ^†^ *p* < .1, *p < .05, **p < .01, ***p < .001.

Graminoid prevalence and environmental drivers had contrasting patterns in terms of the amount of variability explained in the different relatedness metrics (Table A6): graminoid prevalence was the most important driver for relatedness disparity (11% for _SES.MNTD_ and 6% for Δ_SES.MPD_) and the least for nondominant relatedness (5% for ND_SES.MNTD_ and 0% for ND_SES.MPD_), while the opposite was true for the variance explained by environmental variables (8%, 14%, 16%, and 22% for Δ_SES.MNTD_, Δ_SES.MPD_, ND_SES.MNTD_, and ND_SES.MPD_, respectively). Noticeably, as can be seen in these numbers, the relatedness metrics sensitive to tip distances (MNTD) was more affected by graminoid prevalence (a proxy of graminoid competitive performance), while the metric more affected by the basal structure of the tree (MPD) was more affected by environmental variables.

The d‐separation tests indicated that most correlations between predictors and relatedness responses were mediated by graminoid prevalence (Table 2) or required the simultaneous inclusion of two or more variables (not shown). Few variables had a consistent effect not mediated by graminoid prevalence: Diurnal temperature range tended to intensify the negative disparity (*p* < .05), consistent with a stronger facilitation effect in areas with harsher conditions. Grazing tended to decrease dominant clustering besides any effect on graminoid prevalence, consistent with grazers affecting mostly dominant species (*p* < .05).

**TABLE 2:** Tests of the independence of relatedness disparity (Δ_SES.MNTD_, Δ_SES.MPD_), dominant relatedness (D_SES.MNTD_, D_SES.MPD_) and nondominant relatedness (ND_SES.MNTD_, ND _SES.MPD_) from site level descriptors after controlling for graminoid prevalence. Only results with *p* < .1 are shown

Relatedness metric modeled	Independence claims	Parameters of the predictor in italics			
		Estimate	*SE*	Critical value	*p*‐value
Δ_SES.MNTD_	~ *(Grazed)* + PropGram	0.870	0.480	1.812	.075^†^
	~ *(Temperature Range)* + PropGram	−0.138	0.068	−2.036	.046*
D_SES.MNTD_	~ *(Richness)* + PropGram	−0.012	0.006	−1.889	.064^†^
ND_SES.MNTD_	~ *(Biomass)* + PropGram	−0.317	0.176	−1.803	.077^†^
Δ_SES.MPD_	~ *(Annual temperature Range)* + PropGram	0.040	0.022	1.855	.069^†^
D_SES.MPD_	~ *(Grazed)* + PropGram	0.763	0.358	2.132	.037*
	~ *(Richness)* + PropGram	−0.019	0.007	−2.631	.011*

Notes

Each independence claim test the assumption that either Δ_SES.MNTD_, D_SES.MNTD,_ or ND_SES.MNTD_ are not related to the predictor in italics after controlling by graminoid prevalence. Graminoid prevalence measured as the proportion of graminoids of the total biomass (PropGram). All tests had 59 degrees of freedom. Residuals were normally distributed in all the independence claim regressions (*p* > .3, Shapiro–Wilk test). Significance: ^†^ *p* < .1, **p < .01, *p < .05.

## 4 DISCUSSION

In our examination of the interplay between dominant and nondominant grassland species across our globally distributed study, we found key differences in the assembly patterns observed in dominant and nondominant species in communities. Dominant species were more likely to be closely related (i.e., they were more phylogenetically clustered) and share functional traits than nondominant species. As it is implicit in the name, grasslands are often dominated by at least one grass or sedge species, but that name confers little information about the interaction of the site dominant grass with other grasses, and no information about the nature of the nondominant species. Here, we report that a handful of graminoid genera were more likely to share dominance in most sites, while a few other nongraminoid genera shared dominance in other sites. In contrast, the nondominant species were drawn from several lineages, with some lineages more likely to be nondominant than dominant species. The implications and drivers of these findings are reviewed in the following sections.

### 4.1 Different ecological mechanisms drive the assembly of dominant and nondominant species

We found that dominant species were more strongly phylogenetically clustered than were nondominant species, and this negative relatedness disparity was consistent with the trend observed in traits, suggesting a difference in the predominance of the community assembly mechanisms acting on each of these partitions of the community. The results match other findings suggesting fundamental differences in dominant and nondominant species (Arnillas & Cadotte, 2019; Chai et al., 2016; Lennon et al., 2011; Maire et al., 2012; Norden et al., 2017; Ricotta et al., 2008). Here, we review alternative mechanisms that can lead to the negative disparity reported here (see Table A1). First, a negative disparity is consistent with the scenario hypothesized before that environmental constraints have overwhelming effects on the assembly of dominants, while these environmental effects on nondominants are weaker and can be ameliorated by dominants. In particular, the clustering of dominant species suggests that the environment might provide a selective pressure resulting in a single optimal strategy that outperforms other species, a strategy that has been successfully exploited by some graminoids and a few forb lineages (Cadotte & Tucker, 2017; Kraft, Adler, et al., 2015; Mayfield & Levine, 2010; Webb et al., 2002). This holds true even for graminoids, as every site had at least three graminoid species, which makes it possible to have either an overdispersed pattern (one dominant and two nondominant graminoid species), or a more symmetric distribution (one graminoid species per dominance partition). Further, positive interactions provide an alternative mechanism if species from the most common dominant lineage, the graminoids, interact more positively (less negatively) among themselves than with forbs, facilitating the presence of other species in the same lineage compared with species from other lineages (Tables A1‐A3).

We found little support for the scenario that dominants were dissimilar and closely related, whereas nondominants were similar and distantly related (Table A1), as graminoids tend to have very similar structures (shoots and roots), far less variation than among forbs. We cannot rule out the possibility of substantial plasticity and trait divergence at a local scale, or of less conspicuous (or unmeasured) traits playing a key role in community assembly; detailed plot‐level trait measurements are needed to confirm our interpretation.

In contrast to the pattern for dominant species, nondominant species tended to be either random or overdispersed, suggesting four possible processes: (1) a balance between filtering and species interactions, (2) an scenario in which all species are equally likely to become extinct (Tables A1‐A4), (3) a stronger role of species interactions (Weiher et al., 2011; among nondominants or with dominant species), or (4) small‐scale heterogeneity providing diverse niches (Aarssen et al., 2006). As MPD and MNTD provided different patterns, the interpretation of the disparities seems to be related to the evolutionary history of the lineages (see next section). Biogeographic constraints could explain the large among‐site turnover of nondominant species (Figure 2a). However, if biogeographic patterns were important in explaining the relatedness disparity, dominants should be cosmopolitan and nondominants should always have a more restricted range. However, this pattern is not supported by our data because some lineages were nondominant and cosmopolitan (e.g., orchids), while others were dominant despite having a restricted distribution (e.g., goldenrods).

### 4.2 Global drivers of relatedness disparity

We found that most of the global variability in relatedness disparity was mediated by a negative effect of graminoid prevalence. This pattern is consistent with the asymmetric competition model proposed by Mayfield and Levine (2010), in which local environmental conditions (e.g., light) constrain the successful species to a relatively small phylogenetic group (e.g., tallest plants). Identifying the traits shared by a wide variety of species dominant in this environment, like graminoids and goldenrods (*Solidago* spp.), could shed some light on key strategies or properties that allow a species to dominate this environment. While beyond the scope of the current analysis, these shared traits could provide further insights into species invasibility or how nonherbaceous communities could respond when climate changes toward ambient conditions like the ones observed in grasslands.

Environmental variables explained a larger proportion of relatedness metrics based on mean phylogenetic distance (MPD) than of metrics based on mean nearest taxonomic distance (MNTD), while the opposite was true for graminoid prevalence, consistent with the findings of Arnillas and Cadotte (2019). We hypothesize that the MPD and MNTD differences observed were related to different odds of trait differentiation in terms of the number of traits changing and the magnitude of their change, which in turn affect the nature of species co‐existence. First, recent studies suggest that competitive differentiation is mostly associated with fewer traits than niche differences (Cadotte, 2017; Kraft et al., 2015). And second, MNTD is more sensitive to the tips of the phylogeny, while MPD is more affected by the basal part of the tree (Cadotte & Davies, 2016). Further, if we assume that the number of traits changing—not just the magnitude of each trait change—between two species increases with phylogenetic distance, more traits should be involved in the co‐existence mechanisms captured by MPD than by MNTD, and we should expect a switch from competitive to niche differences underlying co‐existence with increasing phylogenetic distance. This expectation is consistent with our finding that graminoid prevalence affected the MNTD‐related metrics more (competitive differentiation), while environment primarily influenced the MPD metrics (niche differentiation). Therefore, the average overdispersion of nondominants using MNTD compared to the average random pattern of MPD is consistent with nondominants dynamic driven by competitive differentiation and not by niche differences.

The nondominant overdispersion using MNTD and random MPD pattern is not consistent with dominant species creating several smaller fragments with heterogeneous environmental conditions, each with different optimal combinations of traits that relatively few species can occupy (Aarssen et al., 2006; Huston, 1994), and niche differentiation with reduced competition should generate the opposite trend (random MNTD, overdispersed MPD). However, it is consistent with dominant species creating a new environment that equalizes the fitness of the nondominant species, allowing species to coexist neutrally regardless of their ecological and trait differences (Chesson, 2000). This equalization is consistent with dominant plants reducing but not depleting the available resources, and even engineering and homogenizing the environment that nondominants occupy (McIntire & Fajardo, 2014; Laland et al., 2016; Arnillas, 2019; cf. Maire et al., 2012).

We found mixed support for the hypothesis that facilitative interactions among distantly related species increases under harsher environmental conditions (Lortie & Callaway, 2006). For instance, negative disparity was positively associated with daily temperature range as expected, but annual temperature range showed the opposite pattern. The average phylogenetic distance between species in the dominant and nondominant partitions may provide further insights into the relatedness disparity between these partitions under different environmental conditions. Further, if local species interactions are more important for MNTD responses, plot‐level descriptors (e.g., soil nutrient availability, soil depth) might explain Δ_SES.MNTD_ variability better than the site descriptors used here. These results need to be thoroughly tested in locally controlled conditions, validated with larger sample size, and other derived implications need to be tested globally also. For instance, relatedness disparity may change with succession when different combinations of nondominants arrive and fill the niche space left by the dominants, and different successional stages in the sample could also explain part of the observed variability (Norden et al., 2012, 2017).

### 4.3 Nondominance as a strategy

The finding that more than a dozen lineages had species with a higher probability of being nondominant than dominant contrasts with classical formulations of theoretical ecology. Some classical life history frameworks, such as r‐K (Reznick et al., 2002) and ruderal‐competitor‐stress tolerant strategies (Grime, 1974), have often been used to identify key traits that would allow a species to become dominant under specific conditions. These widely used frameworks are not explicit with respect to nondominant species’ life histories and suggest that nondominant species are those that could become dominant elsewhere but are found in a suboptimal habitat. Similarly, Rabinowitz (1981) suggested that nondominant (rare) species are either failing, increasing in population size or range, or strongly limited by other species (Gaston, 1994). Specifically, Rabinowitz (1981) argued that “rarity” (which includes nondominance in local conditions, as we use it here) cannot be an “adaptive strategy” because: (1) if successful, the higher fitness of the rare individuals compared to the most common ones should reduce the evolutionary advantage of rarity; and (2) species more likely to be dominant should drive the nondominants to extinction. If this was the case, and if nondominance is a transient state, then no lineage should be more likely to be nondominant than dominant, unless the entire lineage is headed toward extinction or is dominant in a different habitat type. In contrast to this prediction, we found more than a dozen genera in grasslands distributed around the world that are more likely to have nondominant species than dominant ones, many of them with hundreds of herbaceous species and therefore unlikely to be dominant species in nonherbaceous ecosystems (e.g., *Viola*, *Chenopodium*, *Oenothera*, *Verbena*, *Oxalis*). This result is consistent with findings from Amazon rainforests where a few genera in a few families are more likely to be dominant than species in any other genus or family (ter Steege et al., 2013).

We hypothesize that there are sets of traits that result in nondominance as a successful ecological strategy, which would explain why nondominant lineages are geographically widespread and repeatedly occur in the phylogeny. A superb example is the orchids—the family is composed almost entirely of nondominant species in any biome they occupy, yet they grow on every continent and are the most speciose plant family (The Plant List, 2013). Orchids are certainly not following a failing strategy by most objective assessments, yet they are unlikely to have been dominant in any previous geological time. Rather, orchids occupy a unique niche space with a life history that results in nondominance. This observation is consistent with the scenario that dominants and nondominants follow the same assembly rules but that the group of nondominant species is at least partially composed by a different suite of species (Tables A1‐A2). Some theoretical work and experimental results (e.g. Arnillas & Cadotte, 2019; Laland et al., 2016) suggest that assembly rules are indeed different, but more experiments are needed to assess the relative importance of assembly asymmetry and species/lineage identity in driving this observed pattern in grasslands, and to test whether the same trend exists in other biomes.

Nondominant species are often conceptualized as facing a challenging environment dominated by the dominant species, and therefore exploiting marginal conditions, such as growing early in the spring, or just barely persisting in the face of dominant competitors. Further, it is expected that nondominant species face common specific challenges, such as finding viable partners if sexual reproduction is needed (Farnsworth, 2007; Vermeij & Grosberg, 2018). However, nondominant species also can benefit from dominant plants (McIntire & Fajardo, 2014 and references therein) because of the more stable microclimatic environment they produce, reduced pressure from herbivores, pathogens or other negative density‐dependent mechanisms (Aarssen et al., 2006; Rabinowitz et al., 1984), or by diverting resources involved in obtaining and maintaining dominance. A population of nondominant plants with the adequate suite of traits could accumulate or invest in reproduction or seed dispersal with these unused resources. Such populations could thrive and evolve as nondominant as long as a dominant species occupies the same area and provides equivalent benefits.

Nondominance also could have important evolutionary implications—smaller and more isolated populations could increase speciation rates or increase the odds of gene fixation. Because dominant and nondominant species differ in the characteristics of the environment they face and in the restrictions on sexual reproduction, the origins of intraspecific trait variability (genetically driven vs. plasticity) could also differ. More research is required to confirm the suite of traits associated with nondominant species and the importance of their role in the co‐existence and evolution of dominance strategies within herbaceous and nonherbaceous terrestrial habitats.

### 4.4 Future steps

Our study sites are located in herbaceous‐dominated areas around the world (Borer et al., 2014), which frequently include at least one dominant graminoid species, and always have several graminoid species. The range of characteristics of the site floras provides some benefits (e.g., comparability among sites), but also three potential limitations. First, it could be argued that if a lineage in the phylogeny is composed of species more likely to be dominants it would increase the odds of having a clustered pattern (Tables A1‐A5). However, when limiting similarity is strong, a single graminoid should outcompete other graminoids, leaving only one dominant graminoid. This is an extreme case, but the pattern should hold true: strong competition by the dominant graminoid should make the other graminoids less likely to be dominants. Therefore, assuming strong competition positive relatedness disparity should emerge, but we observed negative disparity. Further, the clustered pattern seems driven by a small fraction of the graminoid genera (only 10 out of 128 graminoid genera). The most parsimonious explanation is that the optimal conditions preferred by the dominants correspond to the local environmental conditions and, as a group, they tend to be competitively superior to species with a different set of traits under these conditions (Mayfield & Levine, 2010). Similarly, selection bias cannot explain why the nondominants were more distantly related using MNTD because if graminoids are dominant and a single graminoid species outcompete other graminoids, the resulting nondominants should be as over‐dispersed as the dominant species, and therefore the pattern should appear more random.

Second, grasses and sedges are two groups for which species identification is particularly challenging, resulting in an undercounting of rare species. The failure to detect the presence of low cover graminoids could reduce the strength of the patterns that we found. However, our findings are consistent with similar results in other plant communities (Lennon et al., 2011) and by random patterns observed in communities comprised of species with little capacity to modify their environment (e.g., chironomids Siqueira et al., 2012).

Third, graminoids could interact differently with species within their lineage than with other lineages of plants (Cahill et al., 2008). Taken together, and because some of these arguments cannot be tested using the current dataset, we argue that this framework should be tested in forests, shrublands, and other vegetation types to assess the generality of the negative disparity in plant communities.

Finally, our results clearly suggest an asymmetry between dominant and nondominant species that should be related to differences in reproductive (Vermeij & Grosberg, 2018) as well as in functional traits (Umaña et al., 2017). The preliminary results obtained with traits in this study also point toward an asymmetry between dominant and nondominant species. A more comprehensive trait analysis might also shed some light on the mechanisms driving the observed differences between MPD and MNTD.

## 5 CONCLUDING REMARKS

In contrast with current models that assume a constant set of community assembly mechanisms acting on all species in a community, we found that, in herbaceous plant communities, dominant species show phylogenetic clustering while nondominants show larger phylogenetic dispersion. Preliminary trait analysis also points in the same direction. Previous studies have found differences in population dynamics, and in reproductive and functional traits between dominants and nondominants. Our results suggest two new complementary hypotheses that require additional data to be tested: (1) nondominant species include a set of species not included among the potential dominant species, and (2) the difference between dominant and nondominants is caused by dominant plants ameliorating the effect of the environment on nondominants instead of dominant plants depleting the available resources. We found evidence that species’ dominance tends to be phylogenetically constrained, implying that traits that tend to confer dominance are conserved in the phylogeny and, unexpectedly, that traits that tend to confer nondominance are also conserved. This finding is consistent with the first hypothesis. Our results suggest that dominant and nondominant species benefit from different conditions, with potential implications for ecological and evolutionary dynamics.

## CONFLICT OF INTEREST

None declared.

## AUTHOR CONTRIBUTION

**Carlos Alberto Arnillas:** Conceptualization (lead); Data curation (lead); Formal analysis (lead); Investigation (equal); Methodology (lead); Project administration (equal); Resources (equal); Software (lead); Visualization (lead); Writing‐original draft (lead); Writing‐review & editing (equal). **Elizabeth T. Borer:** Data curation (lead); Funding acquisition (lead); Investigation (equal); Project administration (equal); Resources (equal); Writing‐review & editing (equal). **Eric W. Seabloom:** Data curation (lead); Funding acquisition (lead); Investigation (equal); Project administration (equal); Resources (equal); Writing‐review & editing (equal). **Juan Alberti:** Funding acquisition (equal); Investigation (equal); Resources (equal); Writing‐review & editing (equal). **Selene Baez:** Funding acquisition (equal); Investigation (equal); Resources (equal); Writing‐review & editing (equal). **Jonathon D. Bakker:** Funding acquisition (equal); Investigation (equal); Resources (equal); Writing‐review & editing (equal). **Elizabeth H. Boughton:** Funding acquisition (equal); Investigation (equal); Resources (equal); Writing‐review & editing (equal). **Yvonne M. Buckley:** Funding acquisition (equal); Investigation (equal); Resources (equal); Writing‐review & editing (equal). **Miguel Nuno Bugalho:** Funding acquisition (equal); Investigation (equal); Resources (equal); Writing‐review & editing (equal). **Ian Donohue:** Funding acquisition (equal); Investigation (equal); Resources (equal); Writing‐review & editing (equal). **John Dwyer:** Funding acquisition (equal); Investigation (equal); Resources (equal); Writing‐review & editing (equal). **Jennifer Firn:** Funding acquisition (equal); Investigation (equal); Resources (equal); Writing‐review & editing (equal). **Riley Gridzak:** Formal analysis (supporting); Writing‐review & editing (equal). **Nicole Hagenah:** Funding acquisition (equal); Investigation (equal); Resources (equal); Writing‐review & editing (equal). **Yann Hautier:** Funding acquisition (equal); Investigation (equal); Resources (equal); Writing‐review & editing (equal). **Aveliina Helm:** Funding acquisition (equal); Investigation (equal); Resources (equal); Writing‐review & editing (equal). **Anke Jentsch:** Funding acquisition (equal); Investigation (equal); Resources (equal); Writing‐review & editing (equal). **Johannes M. H. Knops:** Funding acquisition (equal); Investigation (equal); Resources (equal); Writing‐review & editing (equal). **Kimberly J. Komatsu:** Funding acquisition (equal); Investigation (equal); Resources (equal); Writing‐review & editing (equal). **Lauri Laanisto:** Funding acquisition (equal); Investigation (equal); Resources (equal); Writing‐review & editing (equal). **Ramesh Laungani:** Funding acquisition (equal); Investigation (equal); Resources (equal); Writing‐review & editing (equal). **Rebecca McCulley:** Funding acquisition (equal); Investigation (equal); Resources (equal); Writing‐review & editing (equal). **Joslin L. Moore:** Funding acquisition (equal); Investigation (equal); Resources (equal); Writing‐review & editing (equal). **John W. Morgan:** Funding acquisition (equal); Investigation (equal); Resources (equal); Writing‐review & editing (equal). **Pablo Luis Peri:** Funding acquisition (equal); Investigation (equal); Resources (equal); Writing‐review & editing (equal). **Sally A. Power:** Funding acquisition (equal); Investigation (equal); Resources (equal); Writing‐review & editing (equal). **Jodi Price:** Funding acquisition (equal); Investigation (equal); Resources (equal); Writing‐review & editing (equal). **Mahesh Sankaran:** Funding acquisition (equal); Investigation (equal); Resources (equal); Writing‐review & editing (equal). **Brandon Schamp:** Funding acquisition (equal); Investigation (equal); Resources (equal); Writing‐review & editing (equal). **Karina Speziale:** Funding acquisition (equal); Investigation (equal); Resources (equal); Writing‐review & editing (equal). **Rachel Standish:** Funding acquisition (equal); Investigation (equal); Resources (equal); Writing‐review & editing (equal). **Risto Virtanen:** Funding acquisition (equal); Investigation (equal); Resources (equal); Writing‐review & editing (equal). **Marc W. Cadotte:** Conceptualization (supporting); Formal analysis (supporting); Funding acquisition (equal); Investigation (equal); Methodology (supporting); Project administration (equal); Resources (equal); Supervision (lead); Writing‐original draft (supporting); Writing‐review & editing (equal).

### OPEN RESEARCH BADGES

This article has been awarded Open Data, Open Materials Badges. All materials and data are publicly accessible via the Open Science Framework at: https://doi.org/10.5061/dryad.pzgmsbcn710.5061/dryad.pzgmsbcn7.

## Supporting information

Appendix 1 and 2

Supplementary information

## Data Availability

Data and codes are available at: https://doi.org/10.5061/dryad.pzgmsbcn7.
