# Supplementary material for: Opposing community assembly patterns for dominant and nondominant plant species in herbaceous ecosystems globally
Source: Ecol Evol. 2021 Nov 22;11(24):17744–61. doi: 10.1002/ece3.8266 (PMC8717298; doi:10.1002/ece3.8266)
Supplement: Supplementary file 1 — Appendix 1 and 2 [file ECE3-11-17744-s002.doc]

**Opposing community assembly patterns for dominant and
non-dominant plant species in herbaceous ecosystems globally**

**Appendices 1 and 2**

Arnillas, C.A^[[1]](#footnote-1)^., Borer, E.T., Seabloom, E.W., Alberti, J., Baez, S., Bakker, J.D., Boughton, E.H., Buckley, Y.M., Bugalho, M.N., Donohue, I., Dwyer, J., Firn, J., Gridzak, R., Hagenah, N., Hautier, Y., Helm, A., Jentsch, A., Knops, J.M.H., Komatsu, K., Laanisto, L., Laungani, R., McCulley, R., Moore, J.L., Morgan, J.W., Peri, P.L., Power, S.A., Price, J., Sankaran, M., Schamp, B., Speziale, K., Standish, R., Virtanen, R. & Cadotte, M.W.

*Ecology and Evolution 2021* DOI: <https://doi.org/10.1002/ece3.8266>

# Appendix 1. Complementary figures and tables

## Complementary figures

Figure A1: Global distribution of the study sites. In blue, sites with cover information only, in red sites with cover and biomass info. Sites were distributed in North America (41 cover, 33 with biomass info), Europe (12 cover, 7 with biomass info), Oceania (11 cover and with biomass info), South America (8 cover, 5 with biomass info), Africa (3, all with biomass info) and Asia (3, all with biomass info).

Figure A2: Relatedness disparity between dominants and non-dominant plants, and the relatedness of these partitions. The relatedness in each site and partition is the standardized effect size of the mean phylogenetic distance (MPD). The columns represent different ways to measure the dominance of the species. We partitioned the community into two (clear) and three (grey) partitions. Vertical dashed line represents zero (random) assortment, and vertical dotted line represent the limit for an independent site to be considered equal to zero. The distribution for three partitions was tested for normality, when non-normal we tested whether the mean ($\bar{\boldsymbol{x}}$) was lower or higher than 0. If normal, we also tested if the variance (s^2^) was lower or higher than the expected variance (2 for disparity, 1 for relatedness). All tests done at p < 0.05.

Figure A3: Phylogenetic dissimilarities among sites when each site is partitioned in two or three dominance partitions. Two dominance partitions include dominant and non-dominant (species, each with half of the species. Three dominance partitions include also an intermediate dominance, each with a third of the species in the site. The phylogenetic dissimilarity is measured as the multisite Sørensen (SOR – multi) and as the mean pairwise Sørensen dissimilarity (SOR – pairs). In both cases, the indices were decomposed in their turnover (SIM) and nestedness (SNE) fractions. Dashed lines represent the observed value when dominance is assigned base on observed mean species cover per plot, while the densities represent the expected value when the species are randomly distributed in the two or three partitions. Rows 1 and 3 include all sites (All), 2 and 4 exclude Australian sites (nAu), and 3 and 6 exclude sites in North America (nNA).

Figure A4: Details of the lineages with species more likely to be dominant. The numbers in the phylogenetic tree in the top-left of each panel represent the location of the detailed section in the whole tree. Detailed trees are provided for lineages with p < 0.05.

*(*Figure A4*b cont.* Node 9 corresponds to species *Erodium botrys)*

*(*Figure A4*c cont.* Node 17 corresponds to species *Galium_verum*)

Figure A5: Details of the lineages with species more likely to be non-dominant. The numbers in the phylogenetic tree in the top-left of each panel represent the location of the detailed section in the whole tree. Detailed trees are provided for lineages with p < 0.05.

*(*Figure A5 *cont.* Node 9 and 10 correspond to species *Oxalis* spp. *and Viola* spp. respectively*.)*

*(*Figure A5 *cont..* Node 22 corresponds to the species *Taraxacum campyloides.)*

*(*Figure A5 *cont.)*

## Complementary tables

Table A1: Evaluation of the seven assumptions and potential caveats identified by Gerhorld et al. (2015). Each assumption is commented in the context of relatedness disparity (the difference in the phylogenetic relatedness of dominant and non-dominant species), and a potential interpretation of a negative or a positive disparity is presented when relevant.

| **Assumption*** | **Caveat*** | **Comments about the applicability of the assumption/caveat to relatedness disparity** | **Alternative scenario if disparity exists (either positive or negative) and the assumption is wrong** | |
| --- | --- | --- | --- | --- |
|  |  |  | **Negative†** | **Positive** |
| “Phylogenetic dispersion reflects dispersion of phylogenetically conserved functional traits” | “Community assembly processes can select for related dissimilar species (or the inverse)” | If there is no consistent trend between phylogeny and trait dispersion, we should expect no disparity between dominant and non-dominant species. | Dominants are dissimilar and closely related and non-dominants are similar and distantly related.  🡪Competition is stronger than filtering among dominants, and the opposite for non-dominants.  ***A1*** | Dominants are similar and distantly related and non-dominants are dissimilar and closely related.  🡪Competition is stronger than filtering among non-dominants, and the opposite for dominants. |
| “A single ecological function can only be performed by a single trait state or combination of trait states” | “Multiple traits, occurring in multiple lineages, may serve the same function” | Relatedness disparity assumes that different sets of traits are relevant for dominant and non-dominant species. | Any key ecological function related to survival (associated to either competition or filtering) is performed by a combination of traits in dominants in a lineage and by several multiple traits in multiple lineages in non-dominants.  🡪 Dominance is not determined by the traits defining the ecological function, but by a different suit of traits.  ***A2*** | Same ecological function is performed by several multiple traits in dominants in different lineages and by a combination of some traits in few non-dominant lineages.  🡪 Dominance is not determined by the traits defining the ecological function, but by a different suit of traits. |
| “Trait similarity causes enhanced competition” | “Trait similarity might facilitate coexistence” | The question extends to the dominant/non-dominant partition: if similarity facilitates co-existence (either by filtering or facilitated coexistence), are dominants and non-dominants equally sorted?†† | Dominants are similar and related, which facilitates their co-existence. Filtering can also play a role.  Low similarity to dominant species is a possible cause for non-dominance, but cannot explain non-dominant overdispersion if present.  ***A3*** | Non-dominant species are similar and related, which facilitate each other. Filtering can also play a role. |
| “Competition necessarily causes exclusion” (re-interpreted as: *Species interactions necessarily causes exclusions at the species level*) | “Competition *(and facilitation)* may be symmetric and without losers or winners *predictable at the species level*.” | This assumption/caveat is grounded on the comparison between an existent community with a potential species pool. When comparing among dominants and non-dominants the comparison is between existent species only. | Filtering causes dominant species to be clustered. In the absence of consistent species interactions, the more random pattern in the non-dominants indicates weaker filtering acting on them.  ***A4*** | Filtering causes non-dominant species to be clustered. In the absence of consistent species interactions, the more random pattern in the dominants indicates weaker filtering acting on them. |
| “The system is at ‘rest’ such that the process of assembly has played out” | “Not enough time has elapsed to lead to exclusion of species” | No assumption of system at rest: the dominants can be transient, same for non-dominants. |  |  |
| “Habitat filtering and competition are alternative processes, not operating together nor interacting” | “Habitat filtering and competition are independent, increase in parallel or mutually imply each other” | We do not assume that they are negatively correlated, but that one (or the other) can prevail. If both mechanisms have similar effect, we expect that relatedness disparity will be close to 0. |  |  |
| “Community phylogenetic dispersion depends on local and present-day processes only; without such local processes, phylogenetic dispersion is random” | “Community phylogenetic dispersion reflects habitat species pools, not local processes” | Dominant and non-dominant species are present in the same locality, and therefore the dispersion filter is similar for both.  Potential habitat selection bias at the global scale, but not applicable at the local scale.  **A5** |  |  |

Notes: * quoted text obtained as it is from original text, unless otherwise stated; *italics* indicate text added by the authors. † Scenarios compatible with our observed results (negative disparity) are indicated (A1-A5) and discussed in the main text. †† Mayfield & Levine (2010) proposed a model in which this pattern holds, but their model is based on asymmetric competition under a limiting resource (i.e., light), and therefore is consistent with our finding of dominant species being clustered because of a strong environmental filter.

Table A2: Variables used to model the difference in the phylogenetic dispersion of dominants and non-dominant plants and the sources of information used to measure them. Range of observed values is also reported for the independent variables.

| **Variables** | **Definition** | **Source*** | |  |
| --- | --- | --- | --- | --- |
| **Response variables** | | |  |  |
| D_SES.MNTD_ | Phylogenetic dispersion (measured as the standardized effect size of the mean nearest taxonomic distance) of the third most dominant plant species in each site. Dominance defined as the mean cover per plot. | Based on NutNet cover and Qian and Jin (2016) | |  |
| ND_SES.MNTD_ | Phylogenetic dispersion (measured as the standardized effect size of the mean nearest taxonomic distance) of the third least dominant plant species in each site. Dominance defined as the mean cover per plot. | Based on NutNet cover and Qian and Jin (2016) | |  |
| Δ_SES.MNTD_ | Difference in the phylogenetic dispersion of dominants minus non-dominants (D_SES.MNTD_ – ND_SES.MNTD_) | Based on NutNet cover and Qian and Jin (2016) | |  |
| **Climatic variables*** | | | | |
| T | Mean annual temperature  Range: -7.6 – 27.3 ˚C | Worldclim 2 | |  |
| TR | Temperature annual range  Range: 14.6 – 46.6 ˚C | Worldclim 2 | |  |
| DR | Mean diurnal temperature range (mean of monthly range)  Range: 5.1 – 19.6 ˚C | Worldclim 2 | |  |
| P | Total annual precipitation in mm (log transformed)  Range: 5.38 – 7.71 | Worldclim 2 | |  |
| PV | Variance of the monthly precipitation in mm  Range: 11.2 – 111.82 | Worldclim 2 | |  |
| **Location variables** | | | | |
| Elev | Altitude above see level  Range: 0 – 4241 m | Worldclim 2 | |  |
| **Management** | | | | |
| Gz | Is the site subject to grazing?  Range: [0,1] | NutNet description | |  |
| An | Is the site of anthropogenic origin? (e.g., restored)  Range: [0,1] | NutNet description | |  |
| Fr | Is the site subject to prescriptive fire?  Range: [0,1] | NutNet description | |  |
| **Biomass variables** | | | | |
| pGr | Proportion of graminoid biomass in the site  Range: 0.14 – 1 | Based on NutNet biomass | |  |
| BM | Total aboveground biomass in g by m^2^  (log transformed) Range: 3.27 – 7.12 | Based on NutNet biomass | |  |
| LM | Legume aboveground biomass in g by m^2^  (log + 1 transformation) Range: 0 – 4.24 | Based on NutNet biomass | |  |
| **Tree topology and phylogenetic information** | | | | |
| Rec | Proportion of the phylogenetic information of the site tree contained in the site tree tips  Range: 0.45 – 0.74 | Based on NutNet cover and Qian and Jin (2016) | |  |
| S | Number of species present in the site  Range: 13 – 94 | Based on NutNet cover | |  |
| PD | Site level Faith’s Phylogenetic Diversity (Faith, 1992), obtained as the sum of the branches of the site tree.  Range: 1200 – 4238 | Based on NutNet cover and Qian and Jin (2016) | |  |
| MPD | Observed site level Mean Phylogenetic Diversity (Webb *et al.*, 2002)  Range: 220 – 336 | Based on NutNet cover and Qian and Jin (2016) | |  |
| MNTD | Observed site level Mean Nearest Taxonomic Diversity (Webb *et al.*, 2002)  Range: 38 – 131 | Based on NutNet cover and Qian and Jin (2016) | |  |
| Gm | Site level Gamma statistics (Pybus and Harvey, 2000), that measures the temporal distribution of the nodes: negative values indicate deeper nodes, while positive values indicate shallower nodes. Obtained as the mean of a hundred randomly generated binary trees based on the site tree.  Range: -1.15 – 5.75 | Based on NutNet cover and Qian and Jin (2016) | |  |
| Bal | Site level Colless statistics (Mooers and Heard, 1997), that measures the symmetry in the branching pattern (balance): negative values indicate balanced nodes, while positive values indicate unbalanced nodes. Obtained as the mean of a hundred randomly generated binary trees based on the site tree.  Range: 17 – 541 | Based on NutNet cover and Qian and Jin (2016) | |  |

Notes: * All Worldclim 2 (Fick and Hijmans, 2017) variables were included in the model, but discarded because they (1) were correlated among each other or (2) they were poor predictors. Variables listed here but not present in the final model were discarded for similar reasons.

Table A3: Genera observed in the study more likely to be a dominant (a), intermediate -dominant (b) or non-dominant (c).

1. Genera most likely to contain dominant species

| **Family** | **Genus** | **Number of species** | **Number of species and sites per partition** | | | **Probability (proportion of dominants = 1/3)** |
| --- | --- | --- | --- | --- | --- | --- |
|  |  |  | **Dominant** | **Intermediate dominance** | **Non-dominant** |  |
| Asteraceae | *Baccharis* | 4 | 4 | 1 | 0 | 0.045 |
|  | *Hypochaeris* | 4 | 13 | 8 | 2 | 0.025 |
|  | *Solidago* | 12 | 18 | 9 | 2 | 0.002 |
| Cyperaceae | *Carex* | 37 | 31 | 22 | 11 | 0.016 |
|  | *Kobresia* | 4 | 4 | 0 | 0 | 0.012 |
| Fabaceae | *Lespedeza* | 4 | 5 | 0 | 2 | 0.045 |
| Poaceae | *Andropogon* | 5 | 11 | 4 | 0 | 0.002 |
|  | *Anthoxanthum* | 1 | 8 | 3 | 1 | 0.026 |
|  | *Bromus* | 17 | 29 | 14 | 8 | 0.001 |
|  | *Calamagrostis* | 8 | 7 | 2 | 0 | 0.008 |
|  | *Chondrosum* | 4 | 5 | 2 | 0 | 0.045 |
|  | *Diheteropogon* | 2 | 4 | 0 | 0 | 0.012 |
|  | *Elymus* | 9 | 14 | 7 | 4 | 0.020 |
|  | *Festuca* | 14 | 24 | 5 | 6 | 0.000 |
|  | *Holcus* | 1 | 8 | 3 | 0 | 0.009 |
|  | *Panicum* | 24 | 22 | 13 | 3 | 0.003 |
|  | *Poa* | 21 | 29 | 12 | 15 | 0.004 |
|  | *Sporobolus* | 13 | 12 | 3 | 4 | 0.012 |
|  | *Tristachya* | 1 | 3 | 0 | 0 | 0.037 |
| Polemoniaceae | *Phlox* | 4 | 4 | 1 | 0 | 0.045 |

1. Genera most likely to contain intermediate dominance species

| **Family** | **Genus** | **Number of species** | **Number of species per partition** | | | **Probability (proportion of intermediate dominance = 1/3)** |
| --- | --- | --- | --- | --- | --- | --- |
|  |  |  | **Dominant** | **Intermediate dominance** | **Non-dominant** |  |
| Apiaceae | *Zizia* | 2 | 0 | 3 | 0 | 0.037 |
| Caryophyllaceae | *Cerastium* | 6 | 3 | 11 | 5 | 0.029 |
| Asteraceae | *Antennaria* | 7 | 3 | 8 | 1 | 0.026 |
|  | *Saussurea* | 5 | 0 | 4 | 1 | 0.045 |
|  | *Sonchus* | 2 | 0 | 3 | 0 | 0.037 |
| Convolvulaceae | *Ipomoea* | 4 | 0 | 5 | 1 | 0.018 |
|  | *Polymeria* | 3 | 0 | 3 | 0 | 0.037 |
| Equisetaceae | *Equisetum* | 2 | 0 | 3 | 0 | 0.037 |
| Euphorbiaceae | *Acalypha* | 3 | 0 | 3 | 0 | 0.037 |
| Onagraceae | *Epilobium* | 7 | 0 | 7 | 3 | 0.020 |
| Poaceae | *Dactyloctenium* | 3 | 0 | 3 | 0 | 0.037 |
| Ranunculaceae | *Ranunculus* | 12 | 3 | 11 | 5 | 0.029 |
| Solanaceae | *Physalis* | 4 | 1 | 6 | 1 | 0.020 |

1. Genera most likely to contain non-dominant species

| **Family** | **Genus** | **Number of species** | **Number of species per partition** | | | **Probability (proportion of non-dominants = 1/3)** |
| --- | --- | --- | --- | --- | --- | --- |
|  |  |  | **Dominant** | **Intermediate dominance** | **Non-dominant** |  |
| Amaranthaceae | *Chenopodium* | 2 | 0 | 1 | 4 | 0.045 |
| Betulaceae | *Betula*† | 3 | 0 | 0 | 3 | 0.037 |
| Boraginaceae | *Cryptantha* | 3 | 0 | 0 | 3 | 0.037 |
| Brassicaceae | *Brassica* | 2 | 0 | 0 | 4 | 0.012 |
|  | *Descurainia* | 3 | 0 | 0 | 3 | 0.037 |
| Asteraceae | *Bidens* | 1 | 0 | 0 | 3 | 0.037 |
|  | *Gnaphalium* | 4 | 0 | 0 | 4 | 0.012 |
| Fabaceae | *Argyrolobium* | 4 | 0 | 0 | 4 | 0.012 |
| Geraniaceae | *Geranium* | 13 | 3 | 3 | 12 | 0.005 |
| Onagraceae | *Oenothera* | 4 | 0 | 1 | 5 | 0.018 |
| Polygalaceae | *Polygala* | 9 | 0 | 2 | 8 | 0.003 |
| Primulaceae | *Anagallis* | 1 | 0 | 0 | 4 | 0.012 |
|  | *Lysimachia* | 3 | 0 | 0 | 3 | 0.037 |
| Ranunculaceae | *Delphinium* | 4 | 0 | 0 | 5 | 0.004 |
| Sapindaceae | *Acer*† | 3 | 0 | 0 | 3 | 0.037 |
| Ulmaceae | *Ulmus*† | 4 | 0 | 1 | 6 | 0.007 |
| Urticaceae | *Urtica* | 2 | 0 | 0 | 3 | 0.037 |
| Verbenaceae | *Verbena* | 3 | 0 | 0 | 4 | 0.012 |

Table A4: Life-form, number of species and global distribution of the genera more likely to contain non-dominant species in the study. In grey genus composed mostly of trees and/or shrubs.

| **Family** | **Genus** | **Life-form** | **Species in the genus** | **Distribution** | **Source†** |
| --- | --- | --- | --- | --- | --- |
| Amaranthaceae | *Chenopodium* | Herbs, annual or perennial (rarely suffruticose, or small trees) | 170 | Almost worldwide, but most frequent in temperate and subtropical zones | Dinghushan |
| Betulaceae | *Betula* | Trees or shrubs | 60 | Found in the North temperate and the Arctic regions of Europe and Asia and the Andes in South America | Flora of Pakistan |
| Boraginaceae | *Cryptantha* | Annual, biennial, or perennial herbs | 121 | Amphitropic distribution (some species in the Northern and other in the Southern hemisphere). | †† |
| Brassicaceae | *Brassica* | Herbs annual, biennial, or perennial, rarely subshrubs or shrubs, often glaucous. | 40 | Primarily in the Mediterranean region, especially SW Europe and NW Africa; six species in China. | Dinghushan |
|  | *Descurainia* | Herbs annual or perennial, rarely shrubs. | 40 | Mainly in North and South America (30 species) and Macaronesia (seven species), one species a cosmopolitan weed. | IBSC |
| Asteraceae | *Bidens* | Annuals or perennials. | 150-250 | Widespread, especially in subtropical, tropical, and warm-temperate North and South America; ten species (one endemic, one introduced) in China. | IBSC |
|  | *Gnaphalium* | Herbs, annual or perennial. | 80 | Cosmopolitan | Dinghushan |
| Fabaceae | *Argyrolobium* | Herbs, rarely small shrubs. | 130 | North, tropical and South Africa, Madagascar, South of Europe, West of Asia and Indo-Pakistan subcontinent. | Flora of Pakistan |
| Geraniaceae | *Geranium* | Annual or perennial herbs. | 400 | Mainly distributed in the temperate zone of Northern Hemisphere, but also in the tropics. | Flora of Pakistan |
| Onagraceae | *Oenothera* | Annual, biennial or perennial herbs | 125 | Native to North and South America, with some species widely introduced as escapes from cultivation throughout the world. | Flora of Pakistan |
| Oxalidaceae | *Oxalis* | Herbs (subshrubs, shrubs, or vines) | 700 | North America, Mexico, West Indies, Central America, South America, Eurasia, Africa, Atlantic Islands, Indian Ocean Islands, Pacific Islands, Australia; introduced in Bermuda. | FNA |
| Polygalaceae | *Polygala* | Annual or perennial herbs, or shrubs or small trees, rarely small, woody climbers. | 500 | Widespread worldwide | Dinghushan |
| Primulaceae | *Anagallis* | Herbs, annual or perennial, | 20 | North America, Mexico, West Indies (Jamaica), Central America, South America, Eurasia, Africa. | FNA |
|  | *Lysimachia* | Herbs (shrubs), perennial, | 160 | Nearly worldwide; chiefly north temperate, especially Asia. | FNA |
| Ranunculaceae | *Delphinium* | Herbs, perennial, from fasciculate roots or rhizomes. | 300 | North temperate and arctic subtropical and, in Eastern Hemisphere, tropical mountains (South of equator in Africa). | FNA |
| Sapindaceae | *Acer* | Trees or shrubs. | 129 | Widespread in both temperate and tropical regions of Northern Africa, Asia, Europe, and Central and North America | FNA |
| Ulmaceae | *Ulmus* | Deciduous trees | 45 | Northern hemisphere. | Flora of Pakistan |
| Urticaceae | *Urtica* | Herbs, annual or perennial | 45 | Nearly worldwide. | FNA |
| Verbenaceae | *Verbena* | Herbs or subshrubs, annual or perennial. | 250 | Chiefly in tropical America | Dinghushan |
| Violaceae | *Viola* | Herbs, annual or perennial, | 400-600 | Nearly worldwide, temperate regions, also South America, Pacific Islands (Hawaii, Philippines, Taiwan). | FNA |

Notes: † Databases accessed through web portal eFloras ([www.efloras.org](http://www.efloras.org/), accessed on 2019-01-11). †† Genera not available on eFloras, information obtained from <http://www.sci.sdsu.edu/plants/cryptantha/cryptax.html> (accessed on 2019-01-11).

Table S5 Correlation between graminoid prevalence and relatedness disparity, dominance relatedness, and non-dominant relatedness. Relatedness was measured using mean taxonomic nearest distance (Δ_SES.MNTD_, D_SES.MNTD_, ND_SES.MNTD_) and mean phylogeneteic distance (Δ_SES.MPD_, D_SES.MPD_, ND_SES.MPD_).

| **Predictor** | **MNTD** | | | **MPD** | | |
| --- | --- | --- | --- | --- | --- | --- |
|  | **Δ_SES.MNTD_** | **D_SES.MNTD_** | **ND_SES.MNTD_** | **Δ_SES.MPD_** | **D_SES.MPD_** | **ND_SES.MPD_** |
| (Intercept) | 0.449 | 0.023 | -0.426 | 1.427 * | 1.177 * | -0.25 |
| Graminoid prevalence | -2.788 ** | -1.310 * | 1.479 * | -2.938 ** | -2.671 *** | 0.268 |
| R^2^ | 0.138 | 0.083 | 0.103 | 0.147 | 0.205 | 0.003 |

Table S6: Proportion of the variance in relatedness disparity (Δ_SES.MNTD_, Δ_SES.MPD_), dominant (D_SES.MNTD_, D_SES.MPD_) and non-dominant relatedness (ND_SES.MNTD_, ND_SES.MPD_) explained by different categories of drivers. The proportion was estimated as the difference between the final predicted model R^2^ and the R^2^ of a similar model without the variables in each category.

| **Relatedness response variable** | **Variance explained by category of variable** | | | | **Full model R^2^** |
| --- | --- | --- | --- | --- | --- |
|  | **Tree topology and phylogenetic information** | **Environment (location, climate, management)** | **Aboveground biomass** | **Graminoid prevalence** |  |
| Mean nearest taxonomic distance (more sensitive to the tips of the phylogeny) | | | | | |
| Δ_SES.MNTD_ | 0% | 8% | 0% | 11% | 22% |
| D_SES.MNTD_ | 13% | 11% | 0% | 6% | 27% |
| ND_SES.MNTD_ | 0% | 14% | 3% | 5% | 29% |
| Mean phylogenetic distance (more sensitive to the basal part of the phylogeny) | | | | | |
| Δ_SES.MPD_ | 7% | 16% | 0% | 6% | 38% |
| D_SES.MPD_ | 7% | 12% | 0% | 8% | 44% |
| ND_SES.MPD_ | 8% | 22% | 4% | 0% | 30% |

# Appendix 2. Methods details

## Adaptation of the phylogenetic tree

We found that 45.6% of the observed species were not present in Qian and Jin (2016) phylogenetic tree. If the genus was present, (41.2% of the observed species), we looked for the other species of the same genus, found the tip distance (branch length of the species terminal node to the closest node), identified the species closest to the median of the tip distances and included the missing species as a sister species of the species closest to the median. If the genus was absent, the new species was linked to the family node (4.3%).

## Tests of phylogenetic dispersion

This test aimed to assess if dominant relatedness (D_SES.MNTD_), non-dominant relatedness (ND_SES.MNTD_) and dominance disparity (Δ_SES.MNTD_) were different from zero globally, regardless the identity of the species in each site. We assumed each site’s D_SES.MNTD_ value represents an independent observation and used a Shapiro-Wilks normality test first. If the site D_SES.MNTD_ were not normally distributed we used a Wilcoxon signed rank test to decide if the mean of the site D_SES.MNTD_ was lower, equal or higher than zero. If normal, we used a Kolmogorov-Smirnoff goodness-of-fit test (KS-test) to assess if the site D_SES.MNTD_ followed a normal distribution with mean 0 and variance 1, indicating random species sorting. If this KS-test was rejected, we kept the sign of the mean site D_SES.MNTD_ to indicate whether clustering or overdispersion prevails. Then, we removed the mean site D_SES.MNTD_ and did a second KS-test with the same parameters to confirm that the lack of fit was caused only by the mean. If the second KS-test was rejected we recorded if the variance was larger or smaller than 1. We repeated the analysis for ND_SES.MNTD_ and for Δ_SES.MNTD_. In the latter case, we used mean 0 and variance 2, assuming Δ_SES.MNTD_ was the difference of two normally distributed variables with mean 0 and variance 1.

**References**

Faith, D.P. (1992), “Conservation evaluation and phylogenetic diversity”, *Biological Conservation*, Vol. 61 No. 1, pp. 1–10.

Fick, S.E. and Hijmans, R.J. (2017), “WorldClim 2: new 1-km spatial resolution climate surfaces for global land areas”, *International Journal of Climatology*, Vol. 37 No. 12, pp. 4302–4315.

Gerhold, P., Cahill, J.F., Winter, M., Bartish, I.V. and Prinzing, A. (2015), “Phylogenetic patterns are not proxies of community assembly mechanisms (they are far better)”, edited by Venail, P.*Functional Ecology*, Vol. 29 No. 5, pp. 600–614.

Mooers, A.O. and Heard, S.B. (1997), “Inferring evolutionary process from phylogenetic tree shape”, *The Quarterly Review of Biology*, Vol. 72 No. 1, pp. 31–54.

Pybus, O.G. and Harvey, P.H. (2000), “Testing macro–evolutionary models using incomplete molecular phylogenies”, *Proceedings of the Royal Society of London B: Biological Sciences*, Vol. 267 No. 1459, pp. 2267–2272.

Qian, H. and Jin, Y. (2016), “An updated megaphylogeny of plants, a tool for generating plant phylogenies and an analysis of phylogenetic community structure”, *Journal of Plant Ecology*, Vol. 9 No. 2, pp. 233–239.

Webb, C.O., Ackerly, D.D., McPeek, M.A. and Donoghue, M.J. (2002), “Phylogenies and community ecology”, *Annual Review of Ecology and Systematics*, Vol. 33 No. 1, pp. 475–505.

1. E-mail: [carlos.arnillasmerino@mail.utoronto.ca](mailto:carlos.arnillasmerino@mail.utoronto.ca) [↑](#footnote-ref-1)
