# Supplementary material for: Opposing community assembly patterns for dominant and nondominant plant species in herbaceous ecosystems globally
Source: Ecol Evol. 2021 Nov 22;11(24):17744–61. doi: 10.1002/ece3.8266 (PMC8717298; doi:10.1002/ece3.8266)
Supplement: Supplementary file 2 — Supplementary information [file ECE3-11-17744-s001.doc]

**Opposing community assembly patterns for dominant and
non-dominant plant species in herbaceous ecosystems globally**

**Supplementary information: Extended analyses**

Arnillas, C.A.^[[1]](#footnote-1)^, Borer, E.T., Seabloom, E.W., Alberti, J., Baez, S., Bakker, J.D., Boughton, E.H., Buckley, Y.M., Bugalho, M.N., Donohue, I., Dwyer, J., Firn, J., Gridzak, R., Hagenah, N., Hautier, Y., Helm, A., Jentsch, A., Knops, J.M.H., Komatsu, K., Laanisto, L., Laungani, R., McCulley, R., Moore, J.L., Morgan, J.W., Peri, P.L., Power, S.A., Price, J., Sankaran, M., Schamp, B., Speziale, K., Standish, R., Virtanen, R. & Cadotte, M.W.

*Ecology and Evolution 2021* DOI: <https://doi.org/10.1002/ece3.8266>

# Alternative definitions of disparity in phylogenetic dispersion

We used five different ways to estimate the difference on phylogenetic dispersion between dominants and non-dominants using mean-nearest taxonomic distance (MNTD). Here we describe the different ways we did the partitions and the analysis done to compare them. Because the partitions would be composed of different number of species, we estimated the standardized effect size of MNTD (SES.MNTD) for each of the partitions (Δ_SES.MNTD_) in each site. In every case, we pruned the phylogenetic tree to the species present in each site before running the analysis. In that way, the standardized effect sizes for any dominance partition used the same species pool that only includes the species observed in that site at the beginning of the experiment, before nutrients were added to the site. Given that the SES estimates included only the species present, the Δ_SES.MNTD_ is a measure of the difference in how dominant and non-dominant plants’ performance correlate with the environment.

## Partition methods

### Partitions with similar number of species

This method assumed that each partition is composed of a similar number of species. The partition with the most dominant species will be composed of the highest-ranking species. The next partition will be composed by the next higher-ranking species, etc. We define partitions for two and three groups of dominance. Δ_ses_ was then the difference in the phylogenetic dispersion metric between the highest ranking minus the lowest ranking partition. Each site phylogenetic dispersion trend was then classified as either “D_SES_ < ND_SES_”, “D_SES_ > ND_SES_” or “NONE” if Δ_ses_ was significantly less, more or equal to 0, respectively.

### Partitioning by Jenks criteria

We used Jenks’ natural break optimization to find two classes of species, dominant and non-dominant, in each site. Each class composed of species with similar abundance. The optimization algorithm cuts the community using an abundance criterion, minimizing the variability inside each class and maximizing the variability between the classes. The class with the largest abundance values corresponded to the dominant species. Δ_ses_ and the site phylogenetic dispersion trend was then estimated as when partitioning in groups with same number of species. The underlying assumption of this method is that a variance criterion can effectively distinguish biologically meaningful processes.

### Thresholds

Because different sites can have different number of dominant species, we explored several cut values in a community to test the hypothesis that at least in one of their Δ_SES.MNTD_ was higher or lower than zero. We defined dominant partitions in each site as a group of species that include either 10, 20, …, 90% (hereafter cutoffs) of the most dominant species in the site. For each one of these cutoffs we split the community in the dominant (D) and non-dominant partition (ND) assigning every species to one of these partitions.

To decide if the dominant and non-dominant partitions were differently assembled, we estimated Δ_SES.MNTD_ for each combination of site, variable and cutoff. We assumed the Δ_SES.MNTD_ values were normally distributed with variance 2 and, if both partitions were equally dispersed, mean 0. At any cutoff, a value significantly lower (higher) than zero indicated dominants were more clustered (overdispersed) than non-dominants. In the absence of a meaningful way to identify a relevant cutoff for every site, we identify the site disparity trend (either “D_SES_ < ND_SES_”, “D_SES_ > ND_SES_”, “NONE” or “BOTH”) if any difference between dominants and non-dominants was significant in any cutoff smaller or equal to a given threshold. For instance, at a 30% threshold, we assumed “D < ND” if any negative significant difference existed at a 10, 20, or 30% cutoff. If, for instance, “D < ND” at 10% and “D > ND” at 30%, we marked that trend as “BOTH”. The actual value Δ_SES.MNTD_ was then assumed to be the one at which the minimum difference existed, unless there was a significant positive difference only. In that case, we used the maximum value.

As expected, the probability of detecting a difference increased with threshold. We opted for the 50% threshold as a compromise between detection capability and error prone of the criteria (Figure SI1). We found no evidence of both trends (D < ND and D > ND) acting simultaneously.

Figure SI1: Proportion of sites with different disparity trends at different thresholds that capture any partition of species in dominants and non-dominants at a cut-off equal or lower than the given threshold. Each panel represents a different dominance measure. We consider four potential disparity trends: At least in one cut-off lower than the threshold (i) the dominants are less phylogenetically dispersed than non-dominants (D < ND), (ii) the dominants are more phylogenetically dispersed than non-dominants (D > ND), (iii) both conditions hold (D < ND at one cut-off and D > ND at another cut-off) and (iv) none condition hold at any cut-off.

### Extremes

In this method to partition the communities we defined the dominant and non-dominant partitions as the ones that would create a minimum or a maximum Δ_SES.MNTD_. We started with the _SES.MNTD_ values for each site, partition and cutoff generated in the *Thresholds* approach (previous section). Then, we obtained the difference between each combination of the dominant and non-dominant partitions. While doing so, each species was assigned to either the dominant or the non-dominant partition, or to none of them. We choose the minimum and maximum Δ_SES.MNTD_, and estimated their significance. We identified the site trend as either “D_SES_ > ND_SES_”, “D_SES_ < ND_SES_”, “NONE” or “BOTH” according the rules in Table SI1. The final Δ_SES.MNTD_ used to compare with the other partitioning methods was estimated base on the observed trend (Table SI1). We opted to focus in in the min(Δ_SES.MNTD_) unless strong evidence against it existed because the minimum curved seems farther from zero, indicating it is less likely to be randomly generated (Figure SI2).

Table SI1: Criteria used to define the site level disparity trend and the measure of Δ_SES.MNTD_ used to compare with the other partitioning methods.

| **Criteria** | **Both** | **D_SES_ < ND_SES_** | **D_SES_ > ND_SES_** | **None** |
| --- | --- | --- | --- | --- |
| Min(Δ_SES.MNTD_) | < 0 | < 0 | = 0 | = 0 |
| Max(Δ_SES.MNTD_) | > 0 | = 0 | > 0 | = 0 |
| Δ_SES.MNTD_ | Min(Δ_SES.MNTD_) | Min(Δ_SES.MNTD_) | Max(Δ_SES.MNTD_) | Min(Δ_SES.MNTD_) |

Figure SI2: Observed density of the Δ_SES.MNTD_ globally when obtaining the maximum and minimum difference in the dominant and non-dominant phylogenetic dispersion. Each panel represents a different dominance measure.

## Comparison among partition methods

To compare the different partition methods we estimated the correlation between the Δ_SES.MNTD_ obtained in each site with each method and compared the number of sites classified in each trend (Table SI2).

Table SI2: Comparison between the different partition methods in terms of the trends in the partitions detected (a) and the correlation between Δ_SES.MNTD_ values (b). The dominants phylogenetic dispersion can either be lower, equal or higher than non-dominants (D < ND, None, D > ND). Two partition methods (thresholds and extremes) can also detect that a site has a dominant/non-dominant partition which is less than zero and another one that is more than zero simultaneously (Both). The numbers in the trends table (a) indicate the number of sites classified in each trend by each method. The numbers in the correlations table (b) indicate the Pearson’s correlation value (**: p-value < 0.01, ***: p-value < 0.001, n=75 sites).

| a. Trends |  |  |  |  |  |
| --- | --- | --- | --- | --- | --- |
|  | **Eq.rich3** | **Eq.rich2** | **Jenks** | **Thresholds** | **Extremes** |
| Both |  |  |  |  | 2 |
| D<ND | 16 | 20 | 16 | 35 | 52 |
| D>ND |  | 2 |  | 6 | 9 |
| None | 62 | 56 | 62 | 37 | 15 |
|  |  |  |  |  |  |
| b. Correlations | | | | | |
|  | **Eq.rich3** | **Eq.rich2** | **Jenks** | **Thresholds** | **Extremes** |
|  |  |  |  |  |  |
| Eq.rich2 | 0.732 *** |  |  |  |  |
| Jenks | 0.392 *** | 0.314  ** |  |  |  |
| Thresholds | 0.686 *** | 0.785  *** | 0.465  *** |  |  |
| Extremes | 0.738 *** | 0.771  *** | 0.423 *** | 0.807  *** |  |

All the partition methods were positively correlated (p < 0.01, most p < 0.001). Despite it was not the most sensitive partition method; we used the partitioning in groups of similar species richness because it had fewer assumptions.

# Trait analyses

We gathered species trait data from TRY (Kattge *et al.*, 2020) and BIEN (Maitner *et al.*, 2018) databases on 27-11-2020. We also gathered maximum plant height from The Manual of Vascular Plants of Northeastern United States and Adjacent Canada (Gleason and Cronquist, 1991) and the database of the Czech Flora (Pladias, n.d.). Inside the TRY dataset, some leaf traits were measured using different protocols. In those cases, we compared trait values of species for which trait values were available using more than one method. In particular, we found that different ways to measure leaf area were highly correlated (Pearson’s R > 0.72), so we combined them. However, different ways to measure SLA were only moderately correlated (Pearson’s R < 0.65), so we treated each way to measure SLA as a different trait.

We estimated the average trait value per species from each dataset, except for maximum height. For maximum height, we estimated the 90th percentile of the recorded values for each species in each of the two datasets for species with multiple values available. We then tested if the different traits were consistent among datasets for those species present in more than one dataset. We kept only the traits with correlation between datasets larger than 0.6, and those traits for which we have at least 600 different species of the 1823 species included in our dataset. Four traits fulfilled those requirements: leaf dry mass per leaf fresh mass (615 species), leaf nitrogen content per leaf dry mass (676), seed mass (1039) and whole plant height (906). The following analysis were carried independently for each trait.

Similar to the phylogenetic analysis, we estimated the mean pairwise Euclidean distance for dominants and non-dominants independently for each trait. As before, the trait dispersion was estimated independently for dominant and non-dominant species in each site, for several cut-offs (each decile, and the 1/3 and 2/3 quantiles), and using the three ranking criteria used before (cover, frequency, cover presence-only). To estimate the standardized effect size, we used the same approach that we used for the phylogenetic analyses, selecting without replacement from the species available in the site the same number of species observed in each dominance category. We did not consider sites with less than 75% of species represented in the trait database in either dominance category. To identify the dominance category cut-off value, we looked among the previously estimated cut-off values and found a cut-off that could satisfy the following conditions: more than 75% of species trait values reported; closest to 1/3 and 2/3 cut-offs to represent the dominant and non-dominant fraction, respectively; and cut-off value no more than 0.15 units away from the targeted value (1/3 or 2/3). We repeated the same procedure using the 1/2 cut-off for dominant and non-dominants. Dominant partitions were –in general– better represented than non-dominant partitions, but it was not rare to find sites for which non-dominants were better represented in the trait dataset (Table SI3). Only sites with enough information in both partitions were included in further analyses.

Table SI3 Analysis of data completeness. The first column represents the number of sites with at least 75% of species in the dominant (D) or non-dominant partitions (ND). The last three columns indicate the proportion of partitions that were estimated with a cut-off that was less than 0.15 units apart from the targeted cut-off (1/3, 1/2, or 2/3) for either both partitions (Valid D & ND), or only one of them (D or ND).

| **Trait** | **variable** | **Sites** | **Valid D & ND** | **Valid D** | **Valid ND** |
| --- | --- | --- | --- | --- | --- |
| Leaf dry mass per leaf fresh mass | Cover | 40 | 0.54 | 0.74 | 0.66 |
|  | Cover presence-only | 41 | 0.48 | 0.79 | 0.52 |
|  | Frequency | 42 | 0.45 | 0.73 | 0.52 |
| Leaf nitrogen content per leaf dry mass | Cover | 49 | 0.37 | 0.77 | 0.38 |
|  | Cover presence-only | 50 | 0.28 | 0.70 | 0.35 |
|  | Frequency | 51 | 0.32 | 0.71 | 0.35 |
| Seed mass | Cover | 65 | 0.66 | 0.82 | 0.74 |
|  | Cover presence-only | 63 | 0.71 | 0.87 | 0.75 |
|  | Frequency | 61 | 0.75 | 0.92 | 0.79 |
| Whole plant height | Cover | 60 | 0.53 | 0.77 | 0.63 |
|  | Cover presence-only | 57 | 0.50 | 0.78 | 0.62 |
|  | Frequency | 56 | 0.48 | 0.79 | 0.57 |

As with the phylogenetic tests, we estimated the disparity in phylogenetic dispersion between the dominant and non-dominant species for each partitioning, and tested if the difference was likely to be equal to zero (treating each site as a replicate). Given the scarce trait data, fewer sites were included in each trait analysis than in the phylogenetic analysis (Table SI4).

Table SI4 Number of sites with enough information to be included in each analysis using the different dominance criteria. In total, the full dataset was composed of 78 sites for which we have full phylogenetic information.

| **Trait** | **Partitions** | **Cover** | **Frequency** | **Cover**  **presence-only** |
| --- | --- | --- | --- | --- |
| Leaf dry mass per leaf fresh mass | 2 | 19 | 20 | 18 |
|  | 3 | 20 | 18 | 25 |
| Leaf nitrogen content per leaf dry mass | 2 | 13 | 15 | 19 |
|  | 3 | 15 | 18 | 17 |
| Seed mass | 2 | 44 | 47 | 43 |
|  | 3 | 45 | 45 | 43 |
| Whole plant height | 2 | 26 | 25 | 30 |
|  | 3 | 31 | 29 | 34 |

Overall, figures show a trend towards negative disparity, consistent with the phylogenetic analyses (dominants less dispersed than non-dominants; dominants often underdispersed and non-dominants often overdispersed, Figure SI3). Taken together, the four traits considered capture leaf investment strategies (i.e., leaf nitrogen content per leaf dry mass and leaf dry mass per leaf fresh mass), growth form (max plant height) and recruitment strategies (seed mass) that are likely to influence different competitive strategies (Denelle *et al.*, 2020; Díaz *et al.*, 2016). However, compared with the phylogenetic analyses, the observed trend is not as strong. We hypothesize that the difference between both approaches may be caused by fewer sites having enough information to estimate the trait dispersal in each partition (45 or less sites compared to 78 for the phylogenetic analysis), and because each trait estimate was noisier because several species trait values were absent from each site. Further, as discussed in detail in the manuscript, phylogenetic signal is likely to capture a broader pattern of differences among species.

Figure SI3: Global and local tests of trait disparity between dominants and non-dominant plants, and the trait dissimilarity of these partitions. The analysis was repeated for four traits: seed mass (a), whole plant height (b), leaf dry mass per leaf fresh mass (c) and leaf nitrogen content per leaf dry mass (d). Each row represents a trait dissimilarity test (disparity= (dominant SES) – (non-dominant SES), dominant SES, non-dominant SES) and the columns represent different ways to measure the dominance of the species. We partitioned the community into two (clear) and three (grey) partitions (each partition with a similar number of species) and plotted the density of sites with the respective dissimilarity value. The dissimilarity in each site and partition is the standardized effect size of the mean trait distance. For the local tests, vertical dotted lines represent the limit for an independent site to be considered equal to zero. Therefore, the areas beyond the dotted lines indicate the proportion of sites with enough evidence by themselves of a non-random assortment. For the global test, triangles represent the mean value for each partition, vertical dashed lines represent zero (which indicates random assortment) and the expression in the top-left corner indicate if the global dispersion was different from zero or not. For that test, the distribution for the three partitions was tested for normality first. When non-normal, we tested whether the mean ($\bar{\boldsymbol{x}}$) was lower or higher than 0. If normal, we also tested if the variance (s^2^) was lower or higher than the expected variance (2 for disparity, 1 for relatedness). All tests were done at p < 0.05.

| 1. Seed mass | 1. Whole plant height |
| --- | --- |
|  |  |
| 1. Leaf dry mass per leaf fresh mass | 1. Leaf nitrogen content per leaf dry mass |
|  |  |

# References

Denelle, P., Violle, C., DivGrass Consortium and Munoz, F. (2020), “Generalist plants are more competitive and more functionally similar to each other than specialist plants: insights from network analyses”, *Journal of Biogeography*, Vol. 47 No. 9, pp. 1922–1933.

Díaz, S., Kattge, J., Cornelissen, J.H.C., Wright, I.J., Lavorel, S., Dray, S., Reu, B., *et al.* (2016), “The global spectrum of plant form and function”, *Nature*, Vol. 529 No. 7585, pp. 167–171.

Gleason, H.A. and Cronquist, A. (1991), *Manual of Vascular Plants of Northeastern United States and Adjacent Canada*, Vol. 834, New York Botanical Garden Bronx, NY.

Kattge, J., Bönisch, G., Díaz, S., Lavorel, S., Prentice, I.C., Leadley, P., Tautenhahn, S., *et al.* (2020), “TRY plant trait database – enhanced coverage and open access”, *Global Change Biology*, Vol. 26 No. 1, pp. 119–188.

Maitner, B.S., Boyle, B., Casler, N., Condit, R., Donoghue, J., Durán, S.M., Guaderrama, D., *et al.* (2018), “The bien r package: A tool to access the Botanical Information and Ecology Network (BIEN) database”, edited by McMahon, S.*Methods in Ecology and Evolution*, Vol. 9 No. 2, pp. 373–379.

Pladias. (n.d.). “Database of the Czech Flora and Vegetation”, available at: https://pladias.cz/.

1. E-mail: [carlos.arnillasmerino@mail.utoronto.ca](mailto:carlos.arnillasmerino@mail.utoronto.ca) [↑](#footnote-ref-1)
